# Excessive COX‐Prostaglandin Signaling Promotes Cellular Senescence and Impaired Skeletal Muscle Recovery in Aging

**DOI:** 10.1111/acel.70843

**Published:** 2026-11-13

**Authors:** Paul‐Emile Bourrant, Zachary J. Fennel, Elena M. Yee, Robert J. Castro, Patrick J. Ferrara, Acintya Sai Yoganath, Chad M. Skiles, Katsuhiko Funai, Micah J. Drummond

**Affiliations:** ^1^ Department of Nutrition and Integrative Physiology University of Utah Salt Lake City Utah USA; ^2^ Department of Physical Therapy and Athletic Training University of Utah Salt Lake City Utah USA

**Keywords:** aging, cellular senescence, cyclooxygenase, disuse atrophy, skeletal muscle, skeletal muscle recovery

## Abstract

The ability to recover skeletal muscle after periods of disuse declines with age leading to persistent deficits in strength and function that extend beyond muscle mass loss. Here, we investigated whether age‐related alterations in lipid remodeling contribute to impairment in skeletal muscle recovery following disuse. Lipidomic and oxylipin profiling revealed that aged mouse muscle is characterized by sustained phosphatidylcholine and omega‐6‐dependent COX‐prostaglandin signaling during early recovery from disuse atrophy. This persistent lipid mediator response was associated with increased senescence burden, fibrosis, and impaired functional recovery. Pharmacological suppression of COX signaling during reloading in aged mice reduced senescence markers, limited fibrosis, and improved muscle function independent of improved muscle size. Mechanistically, senescent induced macrophages produced COX‐2‐dependent PGE2 and impaired myotube size and fusion through paracrine signaling. Activation of EP2 signaling was sufficient to induce a senescent macrophage phenotype. Importantly, a similar age‐associated dysregulation of oxylipin content was observed in older adults after limb immobilization, supporting the translational relevance of these findings. Together, our results identify sustained COX‐prostaglandin signaling as a key feature of impaired skeletal muscle recovery with aging and suggest that targeted modulation of this pathway may improve muscle recovery in older individuals.

**Trial Registration:** ClinicalTrials.gov identifier: NCT04416191

## Graphical abstract

Aging is associated with persistent COX‐derived prostaglandin signaling during skeletal muscle recovery, which is linked to increased cellular senescence, fibrosis, and impaired muscle regrowth. Importantly, pharmacological inhibition of COX signaling reduces senescence burden and improves muscle recovery in aging.

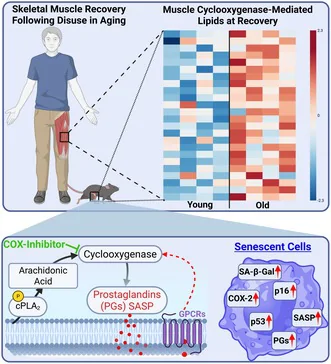

## 1 Introduction

Periods of skeletal muscle disuse, resulting from injury, hospitalization, or surgery, lead to rapid declines in muscle mass and strength (Dirks et al. 2016; Turner et al. 2026; Skiles et al. 2026). While young individuals usually recover skeletal muscle mass and function following these stressors, older adults are less resilient and often experience incomplete recovery, contributing to persistent skeletal muscle weakness, decreased mobility, and a higher risk of disability (Suetta et al. 2013; Bachettini et al. 2020). Importantly, functional impairments in aging are not solely attributable to muscle mass loss, suggesting that maladaptive tissue remodeling and altered cellular composition, such as fibrosis, also play a role in impaired recovery (Larsson et al. 2019; Moiseeva et al. 2023). However, the molecular mechanisms underlying this age‐related inability to restore muscle cellular remodeling and function remain unclear.

Skeletal muscle recovery involves the dynamic remodeling of membrane phospholipids, which serve as structural components and reservoirs for bioactive lipids substrates (Czyzowska et al. 2023). Phosphatidylcholine (PC), one of the most abundant membrane phospholipids, contains esterified polyunsaturated fatty acids (PUFAs), including arachidonic acid (AA), docosahexaenoic acid (DHA), and eicosapentaenoic acid (EPA) (Giannakis et al. 2019). During cellular stress and tissue remodeling, activation of phospholipases, particularly cytosolic phospholipase A_2_ (cPLA2), liberates these fatty acids (Bhattacharya et al. 2009; Pharaoh et al. 2020). These reactions enable their enzymatic or non‐enzymatic conversion into oxylipins, which are diverse bioactive lipid mediators that regulate inflammation, immune cell recruitment, extracellular matrix remodeling, and tissue repair (Markworth et al. 2013; Serhan and Petasis 2011; Markworth, Sugg, et al. 2021). Aging has been associated with altered oxylipin profiles and accumulation of AA‐, EPA‐, and DHA‐derived lipid mediators in skeletal muscle (Czyzowska et al. 2023; Kadoguchi et al. 2023). However, it remains unclear whether membrane lipid remodeling during muscle reloading is disrupted with age and contributes to impaired recovery.

Oxylipins are generated through enzymatic pathways including cyclooxygenase (COX), lipoxygenase (LOX), and cytochrome P450 (CYP), as well as via non‐enzymatic oxidation (Markworth, Brown, et al. 2021). Among these, the COX pathway generates prostaglandins like PGE2 and PGF_2_α, which have well‐established roles in skeletal muscle regeneration, inflammation, and fibrosis (Giannakis et al. 2019; Markworth et al. 2020; Chen et al. 2021). While transient prostaglandin signaling supports acute repair after injury, prolonged or excessive activation may promote maladaptive remodeling (Kim et al. 2016). Aging is associated with altered fatty acid composition and higher levels of omega‐6‐derived lipid mediators in various tissues (Czyzowska et al. 2023; Kadoguchi et al. 2023; Al Saedi et al. 2022). Consistent with this, PGE2 production capacity is increased in aging human skeletal muscle (Liu et al. 2016), suggesting that heightened COX‐prostaglandin activity may contribute to impaired muscle recovery. However, it is not well defined whether age‐related shifts in oxylipin remodeling occur during periods of disuse and reloading and affect functional outcomes.

In parallel, aging skeletal muscle accumulates senescent cells, which adopt a senescence‐associated secretory phenotype (SASP) characterized by pro‐inflammatory and profibrotic signals (Zhang et al. 2022; Cornish and Cordingley 2024). Senescence has been linked to poor recovery, fibrosis, and functional decline in aged skeletal muscle (Xiang et al. 2025; Nelke et al. 2019; Bourrant et al. 2026). Emerging evidence indicates that lipid‐derived mediators can influence stress responses and inflammatory signaling pathways tied to senescence (Wiley et al. 2021; Srour et al. 2024; Cormenier et al. 2018), but the connection between oxylipins and senescence burden during muscle recovery with aging remains to be determined.

Here, we hypothesized that aging alters membrane phospholipid content during muscle recovery, leading to the preferential expansion of omega‐6‐derived and COX‐associated oxylipin pools that contribute to senescence burden and impair functional recovery. To test this hypothesis, we conducted integrated lipidomics and targeted oxylipin profiling in skeletal muscle from young and aged mice subjected to hindlimb unloading and reloading. We investigated how lipid mediator remodeling correlates with senescence burden and muscle recovery, and we assessed the functional impact of pharmacological inhibition of the COX pathway during reloading. Additionally, we investigated the role of downstream prostaglandin signaling in these responses. Finally, we assessed whether age‐related oxylipin remodeling patterns observed in mice are recapitulated in older adults following limb immobilization and recovery. Together, these combined preclinical and clinical studies unmask that prolonged and heightened COX‐associated lipid mediator signaling is linked to increased cellular senescence and impaired skeletal muscle recovery in aging.

## 2 Materials and Methods

### 2.1 Human Study

Young (*n* = 5; 3 males 2 females, 23 ± 3 years, SD) and healthy older adults (*n* = 6; 4 males, 2 females 68 ± 4 years, SD) participants were enrolled from the Salt Lake City area and were part of a larger study published recently (Skiles et al. 2026). Study visits took place at the University of Utah Clinical and Translational Science Institute. All procedures received approval from the University of Utah Institutional Review Board (IRB #130232) and were conducted following the Declaration of Helsinki. Written informed consent was obtained from all participants.

Participants completed 14 days of unilateral limb immobilization, followed by recovery periods of 2 and 7 days (RL2 and RL7). During immobilization, participants wore a knee brace (Orthomen Post‐Op) fixed at 60° of flexion and remained non‐weight‐bearing using crutches or a walker. Skeletal muscle biopsies of the vastus lateralis were obtained after an overnight fast at PRE immobilization, POST immobilization, and after 2 and 7 days recovery (RL2, RL7). Tissue samples (~50 mg) were rapidly frozen in liquid nitrogen and stored at −80°C for subsequent lipidomic analyses. Detailed methods have been described previously (Skiles et al. 2026).

### 2.2 Animals

All animal procedures were approved by the IACUC and conducted in accordance with established guidelines and received official approval (Protocol 1476 to M.J.D.). Mice were housed at the University of Utah Animal Resources Center, an AAALAC‐accredited facility approved by the NIH. All animal experiments were approved by the University of Utah Institutional Animal Care and Use Committee. Young (~4 months) and aged (~25 months) male C57BL/6 mice were sourced from the National Institute on Aging (NIA). The mice were housed under standard conditions with a 12‐h light/dark cycle and had ad libitum access to water and a standard chow diet (Envigo 2920X). In mice, muscle aging is often more pronounced in males, making them a relevant model for studying aspects of human muscle aging (de Jong et al. 2024). While females were not included here, the underlying biological processes are likely shared between sexes and remain important to investigate in future studies (de Jong et al. 2023). Animals were placed into groups with similar starting body weights and were checked daily for general health and weight changes. Any mice that developed signs of illness, marked weight loss, mobility issues unrelated to the study, or tumor burden were removed from the analysis, which were few (~5–8).

### 2.3 Hindlimb Unloading and Reloading

Young and old mice were assigned to one of the following groups: ambulatory controls (AMB), 14 days of hindlimb unloading (HU), or HU followed by 2, 4, or 7 days of recovery (RL2, RL4, and RL7). Control (AMB) mice remained in standard cages (2–4 per cage) and did not undergo unloading. Hindlimb suspension was performed using a modified version of the Morey‐Holton method, with 2 mice per cage during the 14 days of hindlimb unloading (Reidy et al. 2019; Petrocelli et al. 2021; Fennel et al. 2025). Body weight, as well as food and water intake, were monitored daily. Mice in the reloading groups were returned to standard cages for voluntary movement after HU.

At the study endpoint, mice were fasted for 3 h, before euthanasia under isoflurane, followed by cervical dislocation. Hindlimb muscles (gastrocnemius and soleus) were collected, weighed, and either prepared for, snap frozen in liquid nitrogen, or embedded in OCT using chilled isopentane for storage at −80°C.

### 2.4 COX‐Inhibitor Treatment

Following hindlimb unloading (HU), old mice were randomly assigned to one of three treatment groups: (1) low‐dose COX‐inhibitor (30 mg/kg/day), (2) high‐dose COX‐inhibitor (60 mg/kg/day), or (3) vehicle control (5% DMSO in saline). COX‐inhibitor, Ibuprofen (Thermo Scientific #AC258610100) was dissolved in DMSO and saline and administered by intraperitoneal injection using sterile technique. All mice received injections twice daily, once in the morning (~8:00 a.m.) and once in the evening (~5:00 p.m.) for two consecutive days, corresponding to Day 0 (immediately post‐HU) and Day 1 of reloading. Each injection delivered half of the total daily dose (i.e., 15 mg/kg per injection for the low‐dose group and 30 mg/kg per injection for the high‐dose group).

### 2.5 Lipid Peroxidation (MDA) Assay

Malondialdehyde (MDA) levels were measured in gastrocnemius muscle using a lipid peroxidation assay kit (Abcam, ab118970), following the protocol provided by the manufacturer. The absorbance of MDA‐TBA adducts was assessed by measuring absorbance at 532 nm with a spectrophotometer.

### 2.6 Cell Culture and Immortalize Cell Line

Murine C2C12 (CRL‐1772, ATCC, Manassas, VA, USA), NOR‐10 fibroblasts (CCL‐197, ATCC, Manassas, VA, USA), and RAW 264.7 macrophages (TIB‐71, ATCC, Manassas, VA, USA) were cultured in DMEM 4.5 g/dL, 10% FBS, 1% penicillin–streptomycin. For differentiation myotubes DMEM 4.5 g/dL, 2% HS, 1% penicillin–streptomycin. All plated in a 6‐well. Doxorubicin‐induced senescence protocol, cells were treated for 24 h with doxorubicin (DOX, 0.1 μM; Cayman, 15007), or vehicle. Following treatment, cells were washed and maintained in fresh media for 3 additional days, in the absence of experimental treatments. Cells were stained for SA‐β‐Gal, and secretum collected for a PGE2 Elisa (Cayman Item No. 500141).

RAW 264.7 macrophages treatment strategy, cells were treated for 24 h with doxorubicin (DOX, 0.1 μM; Cayman, 15007), DOX + NS‐398 (20 μM; Cayman, 70590), with the EP2 agonist butaprost (1 μM; Cayman, 13740), or vehicle. Following treatment, cells were washed and maintained in fresh media for 3 additional days. Cells were stained for senescence‐associated β‐galactosidase (SA‐β‐Gal) activity using a commercial staining kit (Cell Signaling Technology, #9860) according to the manufacturer's guidelines. Cells were first washed with 1× phosphate‐buffered saline (PBS) and fixed for 15 min at room temperature. After fixation, cells were rinsed twice with 1× PBS before incubation in β‐galactosidase staining solution prepared at pH 6.0 and containing 1 mg/mL X‐gal. Plates were sealed with parafilm and incubated overnight at 37°C in a non‐CO_2_ incubator. Following incubation, the staining solution was removed and replaced with 80% glycerol prior to imaging.

Images were acquired using an inverted Olympus microscope IX71. For each replicate, three non‐overlapping fields were randomly imaged per well, and measurements were averaged to generate a single value per biological replicate. Senescent cells were identified based on positive blue staining, and the percentage of SA‐β‐Gal‐positive cells was calculated relative to the total number of cells in each field.

Triple stain DAPI/p53/COX‐2: RAW 264.7 were fixed in 4% paraformaldehyde (PFA) for 15 min at room temperature, permeabilized with 0.3% Triton X‐100, and subjected to Fc receptor blocking (anti‐CD16/32, clone 2.4G2, BioLegend). To minimize background from mouse primary antibodies, samples were treated with a Mouse‐on‐Mouse (M.O.M. MKB‐2212‐1, VectorLaboratories, Newark, CA, USA) blocking reagent prior to antibody incubation. Cells were incubated overnight at 4°C with mouse anti‐p53 (1:1000, CST 1C12), followed by Alexa Fluor 647‐conjugated anti‐mouse *F*(*ab*′)_2_ secondary antibody (1:500, CST #4410). After additional blocking, samples were incubated with rabbit anti‐COX‐2 (1:500, CST D5H5) overnight at 4°C, followed by Alexa Fluor 488‐conjugated anti‐rabbit *F*(*ab*′)_2_ secondary antibody (1:500, CST #4412). Nuclei were counterstained with DAPI (1:10,000), and cells were maintained in PBS for imaging. Washes were performed in 1× PBS between steps. Images were acquired with a 20× objective using an EVOS FL imaging system (Invitrogen).

For the conditioned media (CM) generation after the 24 h treatment as described above, the media for the 3 days of incubation were adjusted to 2% FBS to limit the effect on myotubes and only capture the effect of the treatment. To maintain differentiation conditions, all CM were supplemented with 2% horse serum (HS) prior to treatment. Fully differentiated myotubes were then exposed to 100% CM from each group for 24 h. Differentiated C2C12 myotubes cultured in six‐well plates were fixed with ice‐cold 4% PFA for 15 min at room temperature. Cells were then washed three times in 1× PBS and permeabilized with 0.1% Triton X‐100 in PBS for 15 min at room temperature.

Following permeabilization, cells were washed and blocked in 2.5% bovine serum albumin (BSA) in 1× PBS for 1 h at room temperature. Cells were then incubated with primary antibody against myosin heavy chain (MF20, DHSB) diluted in blocking buffer (1:100) overnight at 4°C. After washing, cells were incubated with Alexa Fluor 488 secondary antibody (1:250; Invitrogen) for 1 h at room temperature. Nuclei were counterstained with DAPI (5 μM) for 10 min. Cells were washed and maintained in PBS for imaging.

Images were acquired with a 10× objective using an EVOS FL imaging system (Invitrogen). For each replicate, three non‐overlapping fields were randomly selected and imaged per well. Image analysis was performed using MyoCount software (v1.3.1) implemented through MATLAB Runtime (v9.4, R2018a, MathWorks). Myotube area was calculated as the percentage of image area occupied by myosin heavy chain‐positive myotubes. Total nuclei and fusion index were also quantified, with fusion index defined as the proportion of nuclei located within myotubes relative to the total number of nuclei. Myotubes were considered functional when they contained at least three nuclei. Measurements from multiple fields were averaged to obtain a single value per biological replicate.

### 2.7 Grip Strength

Grip strength was evaluated at multiple timepoints (AMB, HU, RL4, RL7) using a grip strength meter (Columbus Instruments, Columbus, OH, USA). Mice were first allowed a short acclimation period (30 s) on the metal grid. Each animal then performed three consecutive grip trials without rest, during which they were gently pulled by the base of the tail. For each trial, the peak force was recorded, and the average of the three trials was used to represent overall grip strength. To maintain consistency, the grip test was performed at the same time of the day across all timepoints, and all four limbs were required to be on the grid before each pull.

### 2.8 Ex‐Vivo Skeletal Muscle Force Production

To assess contractile properties, soleus muscles were harvested from anesthetized mice. Muscles were isolated and secured by tying both tendons in a horizontal in vitro bath system (Aurora Scientific, Model 400A, Ontario, CA) filled with oxygenated Krebs‐Ringer's solution supplemented with 8 mM glucose. A single twitch (20 V, 0.2 ms pulse width) was used to identify the optimal length for force production. Muscles were then allowed to equilibrate before being subjected to a series of tetanic stimulations at increasing frequencies (10–200 Hz, 20 V, 0.2 ms pulse width). All forces were recorded in real time using Aurora Scientific's Dynamic Muscle Control software (DMC v5.500) and analyzed with Dynamic Muscle Analysis software (DMA v5.321). Specific force (mN/mm^2^) was calculated by estimating the muscle cross‐sectional area based on muscle length and wet weight.

### 2.9 Immunofluorescence and Histology

The gastrocnemius and soleus muscles were embedded in optimal cutting temperature (OCT) compound and snap‐frozen in 2‐methylbutane chilled with liquid nitrogen. Tissues were cryosectioned at 10 μm using a Leica CM1860 cryostat. Following staining, sections were imaged using a Zeiss Axio Scan.Z1 slide scanner equipped with a 20× objective lens.

Senescence‐associated β‐galactosidase (SA‐β‐Gal) staining with Hematoxylin and Eosin counterstaining was performed as previously described (Bourrant et al. 2026; Fennel et al. 2025). Briefly, muscle sections were fixed for 10 min in 25% glutaraldehyde, incubated with X‐gal staining solution (pH 6.0) at 37°C, counterstained, dehydrated, and mounted with Cytoseal. SA‐β‐Gal^+^ cells were quantified using Fiji and normalized to section area using QuPath. (Bankhead et al. 2017).

To assess satellite cells (PAX7^+^), sections were fixed with 4% PFA, followed by antigen retrieval in sodium citrate buffer at 92°C. After blocking in 3% hydrogen peroxide, sections were treated with Mouse‐on‐Mouse IgG blocking reagent (Vector) for 1 h. Slides were incubated overnight at 4°C with a primary antibody against Pax7 (1:100, DSHB). The next day, sections were incubated with a biotinylated goat anti‐mouse secondary antibody (Jackson ImmunoResearch), followed by Tyramide SuperBoost kit with Alexa Fluor 488 (Invitrogen). Nuclear and membrane staining was performed with DAPI and wheat germ agglutinin (WGA‐647, Invitrogen). Slides were mounted with Vectashield and stored at 4°C. Satellite cells were identified as PAX7^+^/DAPI^+^ cells, as previously described (Bourrant et al. 2026; Fennel et al. 2025; Fix, Mahmassani, et al. 2021).

γH2AX^+^ cells were visualized using immunofluorescence according to the manufacturer's protocol (Cell Signaling) and as previously described (Bourrant et al. 2026). Briefly, sections were fixed in 4% PFA and washed in PBS. Non‐specific binding was blocked using a solution of 5% goat serum and 0.3% Triton X‐100 in PBS for 1 h. Sections were then incubated overnight at 4°C with an antibody against γH2AX (1:400, Cell Signaling #2577S). After PBS washes, slides were treated with Alexa Fluor 647‐conjugated goat anti‐rabbit secondary antibody (1:1000, Invitrogen) for 90 min. Nuclear and membrane staining was performed using DAPI and WGA‐488 (Invitrogen). γH2AX^+^/DAPI^+^ cells were quantified using Qupath (Bankhead et al. 2017).

To assess cellular senescence, we performed p21^+^ staining, sections were fixed in 4% PFA and washed in PBS. Blocked in 3% hydrogen peroxide for 10 min. Non‐specific binding was blocked with 2% BSA and 0.1% Triton X‐100 for 1 h. Slides were incubated overnight at 4°C with anti‐p21 primary antibody (1:200, Abcam ab109199). The following day, sections were treated with biotin‐conjugated anti‐rabbit secondary antibody (1:1000, Jackson ImmunoResearch), followed by 60 min in streptavidin‐HRP and 15 min in Alexa Fluor 555 (1:200, Invitrogen). After PBS washes, DAPI and WGA‐488 were applied for nuclear and membrane counterstaining. Slides were mounted in Vectashield and stored at 4°C. p21^+^ cells were quantified using Qupath (Bankhead et al. 2017).

To evaluate collagen content, Sirius Red staining was performed; muscle sections were fixed in Bouin's solution (Sigma‐Aldrich) for 1 h at 56°C. Sections were stained with Sirius Red solution (Abcam ab246832) for 1 h at room temperature. Excess dye was removed by washing three times in 0.5% acetic acid and water. Tissues were dehydrated in ethanol, dipped in xylene, and coverslipped with Cytoseal XYL (Fisher Scientific). The area of Sirius Red staining was quantified using FIJI/ImageJ by applying color thresholding to regions of interest.

Cross‐sectional area, frequency of centrally nucleated fibers, and satellite cell content were quantified using high‐throughput automated analysis in FIJI/ImageJ with the MuscleJ version 1.0.2 plugin.

Senescence markers quantification was performed using QuPath (Bankhead et al. 2017). All imaging and measurements were completed blinded to the experimental groups.

### 2.10 Real‐Time PCR

RNA was isolated from gastrocnemius muscle (~20 mg) using QIAzol (Qiagen, #79306), separated and precipitated with chloroform and isopropanol as instructed by the manufacturer. RNA was resuspended in nuclease‐free water and quantified (Take3, BioTek, Winooski, VT). One microgram of RNA was used to reverse transcribe by using the iScript cDNA synthesis kit (Bio‐Rad, #17088‐91) and Bio‐Rad T100 Thermal Cycler (volume 20 μL, 25°C 5 min, 46°C 20 min, 95°C 1 min, 4°C). Real‐time quantitative PCR was carried out using cDNA diluted 1:8 in nuclease‐free water, on a CFX Connect Real‐Time PCR Detection System (Bio‐Rad), with SsoAdvanced Universal SYBR Green Supermix (Bio‐Rad, #17252‐70). Expression levels were adjusted relative to ribosomal protein L32. Fold changes compared to control samples (Saline group) were calculated using the delta–delta *Ct* (2^−ΔΔ*Ct*^) method. We chose gene expression readouts that were representative of senescence and COX‐2 related markers as used by us and others (Moiseeva et al. 2023; Bourrant et al. 2026; Ho et al. 2017). Primer sequences (Table 1) were purchased from the University of Utah Health Sciences Center Core.

**TABLE 1:** RT‐qPCR primer sequences.

Gene	Forward primer (5′‐3′)	Reverse primer (5′‐3′)
L32	TTCCTGGTCCACAATGTCAA	GGCTTTTCGGTTCTTAGAGGA
P53	TGAAAATGTCTCCTGGCTCAG	CTAGCATTCAGGCCCTCATC
p21	CCAGGCCAAGATGGTGTCTT	TGAGAAAGGATCAGCCATTGC
p16INKA	CATCTGGAGCAGCATGGAGTC	ATCATCATCACCTGAATCGGGG
PAI‐1	CCGATGGGCTCGAGTATGA	TTGTCTGATGAGTTCAGCATCCA
Igfbp4	TGAGAGCGAACATCCCAACAA	TGTCCCCACGATCTTCATCTT
Il‐1b	CCAAAATACCTGTGGCCTTGG	GCTTGTGCTCTGCTTGTGAG
Il‐6	GGTGACAACCACGGCCTTCCC	AAGCCTCCGACTTGTGAAGTGGT
IFNɣ	AGCGGCTGACTGAACTCAGATTGTAG	GTCACAGTTTTCAGCTGTATAGGG
TNFα	CGCTCTTCTGTCTACTGAACTT	GATGAGAGGGAGGCCATT
Ccl2	CACTCACCTGCTGCTACTCA	GAGCTTGGTGACAAAAACTACAGC
Ccl8	ACGCTAGCCTTCACTCCAAA	GTGACTGGAGCCTTATCTGG
Cxcl10	TGCCCACGTGTTGAGATCAT	AAGGAGCCCTTTTAGACCTTTT
Timp2	TGCAATGCAGACGTAGTGAT	ATAGATGTCATTCCCGGAAT
Col3a1	TGACTGTCCCACGTAAGCAC	GAGGGCCATAGCTGAACTGA
Ptges‐1	AGCACACTGCTGGTCATCAA	CAGCCTCATCTGGCCTGT
Ptger1	GTGGTGTCGTGCATCTGCT	CCGCTGCAGGGAGTTAGAGT

### 2.11 Oxylipins Profiling

Flash‐frozen skeletal muscle (~20 mg) from human quadriceps and mouse gastrocnemius was analyzed at the University of California San Diego Lipidomics Core for targeted oxylipins quantification. Analyses followed established Core protocols (Quehenberger et al. 2018, 2010) involving solid‐phase extraction, liquid chromatography, and tandem mass spectrometry. Concentrations were determined by stable isotope dilution and normalized to tissue wet weight (pmol mg of tissue).

Skeletal muscle tissue (~20 mg) was homogenized in phosphate‐buffered saline containing 10% methanol and spiked with deuterated oxylipins internal standards. Lipids were isolated by solid‐phase extraction using Strata‐X cartridges (Phenomenex, Torrance, CA). Oxylipins were eluted with methanol, dried under vacuum, and reconstituted in aqueous buffer containing acetonitrile and acetic acid.

Reverse‐phase ultra‐performance liquid chromatography (UPLC) was performed on a RP C18 BEH Shield column (2.1 × 100 mm; 1.7 μm; Waters). Data were collected into an AB SCIEX 6500 QTrap mass spectrometer (IonDrive Turbo V source) operating in negative electrospray ionization mode. Multiple reaction monitoring (MRM) transitions were monitored for each oxylipin, and identities were confirmed by matching retention times and MRM signatures with authentic standards. Absolute quantification was achieved by comparing internal standard peak area ratios to calibration curves prepared under identical conditions. Data are reported as pmol/mg tissue weight.

### 2.12 Mass Spectrometry

All lipidomics experiments were conducted by the University of Utah Metabolomics Core. Lipids were extracted from frozen gastrocnemius muscle tissue using internal standards (Avanti Polar Lipids) following a modified methyl‐tert‐butyl ether (MTBE) extraction protocol. Tissue was weighed and homogenized in a methanol/PBS/internal standard mixture. MTBE was added, and samples were incubated on ice for 1 h with periodic vortexing. Following centrifugation at 15,000 × *g* for 10 min at 4°C, the organic phase was collected and dried using a SpeedVac. Lipid abundance was normalized to tissue weight (mg).

Lipid extracts were analyzed using an Acquity UPLC CSH C18 column with a VanGuard precolumn, maintained at 65°C. Chromatographic separation was performed on an Agilent 1290 UPLC system coupled to a 6545 Q‐TOF mass spectrometer operating in either positive or negative ionization mode. Samples were randomized and scanned over an m/z range of 100–1700. Mobile phase A consisted of ACN:H_2_O (60:40, v/v) with 10 mM ammonium formate and 0.1% formic acid, while mobile phase B consisted of IPA:ACN:H_2_O (90:9:1, v/v/v) with the same modifiers. A flow rate of 0.4 mL/min was maintained, with injection volumes of 2 μL for positive mode and 10 μL for negative mode. Tandem MS was performed using iterative exclusion with collision energies of 20 V and 27.5 V in positive and negative modes, respectively.

Data were processed using Agilent MassHunter Workstation (Qualitative and Quantitative modules). Quality control (QC) samples (*n* = 8) and process blanks (*n* = 4) were included throughout the run to assess reproducibility and contamination. Lipid identification was based on accurate mass and MS/MS spectral matching using the Agilent Lipid Annotator library and LipidMatch. Data from both ionization modes were merged by lipid class. Features were filtered in Excel to include only those with a relative standard deviation (RSD) < 30% in QC samples and a background signal in blanks < 30% of the QC signal. Final lipid intensities were normalized to class‐specific internal standards, tissue weight, and total lipid abundance prior to statistical analysis.

### 2.13 Western Blot

Approximately 30 mg of gastrocnemius muscle tissue was homogenized in lysis buffer (50 mM Tris–HCl pH 7.5, 150 mM NaCl, 5 mM EDTA, 1% Triton X‐100, 0.1% sodium deoxycholate, 0.1% SDS, 1× protease and phosphatase inhibitor) (ThermoFisher). Lysates samples were centrifuged, and the supernatant was collected for analysis. Protein concentration was measured using a BCA assay (Thermo Scientific). Equal amounts of protein were separated on 4%–20% gels and transferred onto a nitrocellulose membrane. Membranes were blocked for 1 h at room temperature in 2.5% milk prepared in TBST, followed by overnight incubation at 4°C with primary antibodies (1:1000) diluted in 2.5% milk‐TBST. The next day, membranes were incubated with secondary antibodies (1:4000) in 2.5% milk for 1 h at room temperature. Protein detection was carried out using ECL Prime chemiluminescent reagent (GE Healthcare, cat #RPN2236), and membranes were imaged on a ChemiDoc XRS system (Bio‐Rad). Band intensities were quantified using Image Lab Software (Bio‐Rad, Hercules, CA, USA). The following primary antibody was purchased from Abcam: Total OXPHOS Rodent #ab110413.

## 3 Results

### 3.1 Aging Impairs Skeletal Muscle Recovery and Alters Membrane Lipid Remodeling Following Disuse

To examine the impact of advanced age on skeletal muscle recovery after a period of disuse, young and old mice underwent 14 days of hindlimb unloading followed by reloading for 2, 4, or 7 days (Figure 1A). Hindlimb unloading induced a loss in whole‐body grip strength in both age groups, however, aged mice failed to fully recover their strength back to baseline levels after 7 days of reloading. (Figure 1B).

**FIGURE 1:**
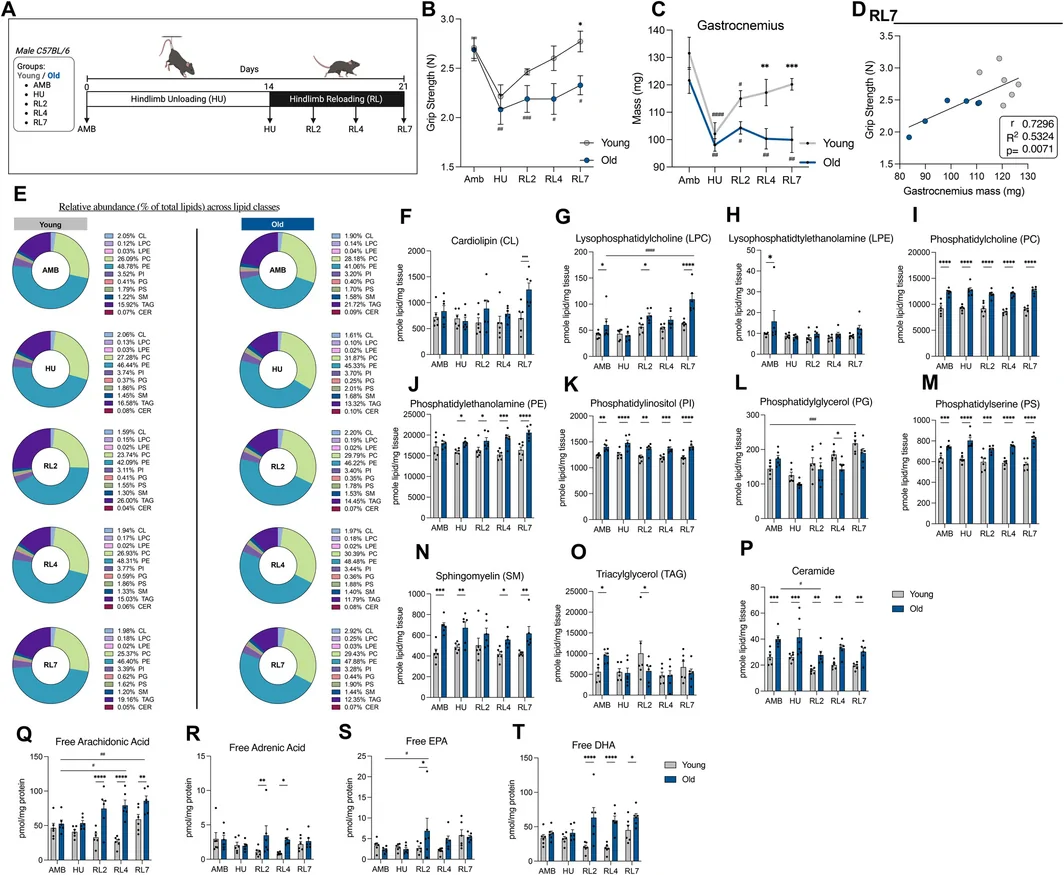
Aging impairs skeletal muscle recovery and drives age‐dependent lipid redistribution following disuse. (A) Experimental design for hindlimb unloading and reloading. Young (~4 months) and old (~25 months) male mice were assigned: ambulatory baseline (AMB), 14 days of hindlimb unloading (HU), and reloading for 2, 4, or 7 days (RL2, RL4, RL7). (B) Longitudinal measurements of whole‐body grip strength in young and old mice at AMB, HU, RL2, RL4, and RL7 (*n* = 6/group and timepoint). (C) Gastrocnemius muscle mass, measured in young and old mice at AMB, HU, RL2, RL4, and RL7. Data represent biologically independent muscle samples (*n* = 6/group and timepoint). (D) Correlation analysis of whole‐body grip strength and gastrocnemius muscle at RL7. Pearson's correlation, with corresponding *p*‐values, *r* and *R* ^2^ displayed on the graph (*n* = 6/group and timepoint). (E) Pie charts represent the relative abundance (% of total lipids) across lipid classes in young and old mice at AMB, HU, RL2, RL4, and RL7. Percent values reflect the mean lipid class distribution calculated from biologically independent muscle samples (*n* = 6/group and timepoint). (F–P) Bar graphs display the total abundance of each lipid class, Cardiolipin (CL), Lysophosphatidylcholine (LPC), Lysophosphatidylethanolamine (LPE), Phosphatidylcholine (PC), Phosphatidylethanolamine (PE), Phosphatidylinositol (PI), Phosphatidylglycerol (PG), Phosphatidylserine (PS), Sphingomyelin (SM), Triacylglycerol (TAG), and Ceramide (CER), in young and old mice across all timepoints (*n* = 6/group and timepoint). (Q–T) Bar graphs display the total abundance of Arachidonic acid and Adrenic Acid, Eicosapentaenoic acid (EPA) and Docosahexaenoic acid (DHA) (*n* = 6/group and timepoint). Statistical analysis: All data presented as mean ± SEM. Statistical analyses were performed using two‐way ANOVA followed by Tukey's multiple‐comparisons test. *p < 0.05, **p < 0.01, ***p < 0.001, and ****p < 0.0001. (*) indicates a group difference at a specific time point. #*p* < 0.05, ##*p* < 0.01, ###*p* < 0.001, and ####*p* < 0.0001. (#) indicates a within group difference from their baseline time point. Correlations were evaluated using Pearson's correlation.

Consistent with these functional deficits, we focused subsequent analyses on the gastrocnemius muscle, which displays a mixed fiber‐type composition and consistently exhibits age‐related impairments in recovery following hindlimb unloading (Reidy et al. 2019; Fennel et al. 2025). Gastrocnemius muscle mass decreased after unloading and failed to fully recover in aged mice, whereas young mice regained muscle mass toward baseline levels during reloading (Figure 1C). Similar trends were observed across other plantar flexor muscles, including the soleus and plantaris, while dorsiflexor muscles (tibialis anterior [TA] and extensor digitorum longus [EDL]) were relatively unaffected by disuse and recovery (Figure S1A–D). At RL7, larger gastrocnemius muscle mass correlated positively with greater grip strength in young mice, while aged mice showed persistent impairments in both measures (Figure 1D). Similar positive relationships were found for the soleus and plantaris, which also had significant correlations between muscle mass and grip strength. In contrast, the TA and EDL muscles did not show significant correlations with grip strength (Figure S1E).

To determine whether membrane lipid remodeling accompanied these deficits, we performed untargeted lipidomic profiling of the gastrocnemius muscle across all timepoints (AMB, HU, RL2, RL4, RL7) and groups (young and old). Principal component analysis (PCA) of the gastrocnemius lipidomic data revealed two partially distinct age‐associated clusters already evident at baseline. Following disuse and especially during reloading, these clusters became more clearly separated with reduced overlap, indicating that young and old muscles diverge further in their lipidomic response during reloading (Figure S1F). Relative lipid class abundance (% of total lipids) revealed that phosphatidylethanolamine (PE) and triacylglycerols (TAG) were the main lipid classes across groups, while PC displayed a marked age‐dependent redistribution. In young muscle, PE made up approximately 42%–48% of the lipid pool across timepoints, while TAG ranged from 15% to 26%, and PC ranged from 23% to 27%. In contrast, old muscle showed significant increases in multiple phospholipids. (Figure 1E).

Consistent with membrane remodeling during recovery, LPC gradually increased from RL2 through RL7, while PC abundance remained high in old muscle across all time points (Figure 1G,I). Unlike previous skeletal muscle injury models, in which LPC elevation is linked with PC depletion (Giannakis et al. 2019), our data show that PC levels remain elevated despite increased LPC production, indicating enhanced phospholipid turnover rather than membrane loss. Since PC is a major reservoir of esterified PUFAs, we next investigated the levels of free PUFA species during recovery.

Most membrane PC species contained AA as well as DHA, and EPA. Free AA displayed a pronounced age‐dependent increase at reloading and remained elevated throughout reloading, whereas young muscle maintained relatively low levels at all timepoints (Figure 1Q). In contrast, omega‐3 substrates showed only modest changes, with free DHA mainly differing at reloading stages rather than steadily increasing, and free EPA and free adrenic acid remaining mostly stable (Figure 1R–T). These results suggest that membrane phospholipid remodeling during recovery preferentially mobilizes AA in aged skeletal muscle rather than reflecting a global release of PUFAs. Therefore, this remodeling appears characterized by sustained PC availability alongside increased lysophosphatidylcholine (LPC) and greater release of AA‐containing phospholipids, aligning with increased phospholipase activity and a larger pool of substrates for downstream synthesis of bioactive lipid mediators.

Additionally, correlation analyses revealed a significant inverse relationship between gastrocnemius muscle mass and various membrane lipid classes at RL7, including PC, LPC, and cardiolipin (CL; Figure S1G). Finally, mitochondrial OXPHOS protein levels and lipid peroxidation showed limited age‐related differences during recovery, with only baseline Complex V enrichment in young gastrocnemius muscle and HU‐related increases in MDA in old gastrocnemius muscle (Figure S2A–D). Together, these results further suggest that membrane phospholipid remodeling and cPLA2‐mediated PUFA release and their accumulation are closely linked to impaired skeletal muscle recovery after disuse in aging.

### 3.2 Aging Disrupts Oxylipin Pathway Remodeling During Skeletal Muscle Recovery

Lipid mediators generated from PUFAs are central regulators of inflammatory and tissue remodeling processes in skeletal muscle. To examine how these mediators change during disuse and reloading, we performed targeted liquid chromatography–tandem mass spectrometry (LC–MS/MS) oxylipin profiling of gastrocnemius muscle collected at AMB, HU, RL2, RL4, and RL7.

Targeted oxylipin profiling revealed age and time‐related changes in lipid mediator content, which became even more pronounced during the reloading phase, particularly at RL7 (Figure 2A–I). Analysis of fatty‐acid‐derived pools showed that omega‐3‐derived mediators exhibited age differences primarily detected at RL2 and RL4, but without sustained elevations at RL7 (Figure 2A). In contrast, omega‐6‐derived oxylipins displayed an earlier onset and more persistent increase in aged muscle, becoming significantly elevated relative to young muscle at RL2 and RL4 and remaining higher at RL7. Within‐group comparisons further indicated that omega‐6 levels in old muscle were significantly increased relative to their ambulatory baseline by RL7, whereas young muscle did not exhibit significant changes (Figure 2B).

**FIGURE 2:**
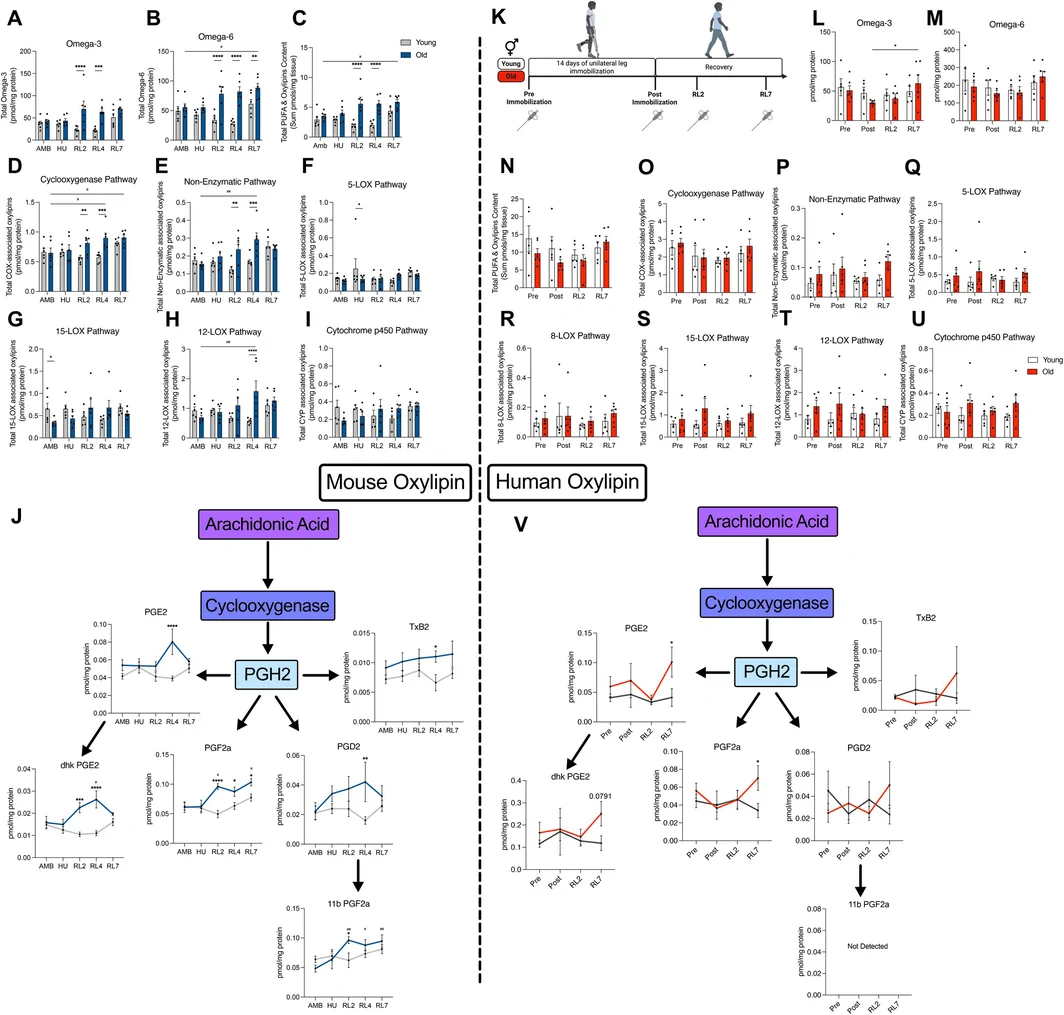
Oxylipin signaling shifts in human skeletal muscle during immobilization and recovery, with mirrored responses in mice. (A–I) Bar graphs show total omega‐3 and omega‐6 derived lipids as well as, total PUFA & oxylipins content and pathway‐specific subsets including, total cyclooxygenase associated oxylipins (COX), total non‐enzymatic associated oxylipins, total 5‐lipoxygenase associated oxylipins (5‐Lox), total 15‐lipoxygenase associated oxylipins (15‐LOX), total 12‐lipoxygenase associated oxylipins (12‐LOX), and total cytochrome P450 associated oxylipins (CYP), in gastrocnemius muscle of young and old mice at AMB, HU, RL2, RL4, and RL7 (*n* = 6/group and timepoint). (J) Line graphs of individual arachidonic acid COX‐derived prostaglandins (PGE2, dhk PGE2, PGF2α, PGD2, 11b PGF2α and TxB2) in young and old mouse gastrocnemius skeletal muscle at AMB, HU, RL2, RL4, and RL7 (*n* = 6/group and timepoint). (K) Human experimental design of unilateral leg immobilization. Young (*n* = 5; 3 males 2 females, 23 ± 3 years, SD) and healthy older adults (*n* = 6; 4 males, 2 females 68 ± 4 years, SD) participated in 14 days of unilateral leg immobilization, followed by 2 and 7 days of recovery. Quadriceps muscle biopsies were collected during Pre immobilization (Pre), Post 2 weeks immobilization (Post), 2 days of recovery (RL2) and 7 days of recovery (RL7). (L–U) Bar graphs show total omega‐3 and omega‐6 derived lipids as well as total PUFA & oxylipins content and pathway‐specific subsets including total cyclooxygenase‐associated oxylipins (COX), total non‐enzymatic associated oxylipins, total 5‐lipoxygenase‐associated oxylipins (5‐LOX), total 8‐lipoxygenase‐associated oxylipins (8‐LOX), total 15‐lipoxygenase‐associated oxylipins (15‐LOX), total 12‐lipoxygenase‐associated oxylipins (12‐LOX), and total cytochrome P450‐associated oxylipins (CYP) in vastus lateralis muscle biopsies from young and older adults before immobilization (Pre), following 14 days of immobilization (Post), and after 2 and 7 days of recovery (RL2 and RL7) (*n* = 5–6/group and timepoint). (V) Line graphs of individual arachidonic acid COX‐derived prostaglandins (PGE2, dhk PGE2, PGF2α, PGD2, 11b PGF2α, and TxB2) in young and old healthy adults' skeletal muscle at Pre, Post, RL2, and RL7 (*n* = 5–6/group and timepoint). Statistical analysis: All data presented as mean ± SEM. Statistical analyses were performed using two‐way ANOVA followed by Tukey's multiple‐comparisons test. *p < 0.05, **p < 0.01, ***p < 0.001, and ****p < 0.0001. (*) indicates a group difference at a specific time point. #*p* < 0.05, ##*p* < 0.01, ###*p* < 0.001, and ####*p* < 0.0001. (#) indicates a within group difference from their baseline time point.

Consistent with this, total oxylipin abundance increased significantly in aged muscle beginning at RL2, and old muscle demonstrated a significant elevation at RL7 compared with its own baseline, while young muscle remained comparatively stable across timepoints (Figure 2C). Pathway‐specific analysis identified significant age differences in COX‐associated oxylipins at RL2 and RL4 and significant increases from baseline within old muscle at RL4 and RL7, whereas young muscle displayed no significant increase (Figure 2D). The non‐enzymatic oxylipin pool followed a similar but less sustained trajectory, with significant age differences emerging at RL2 and RL4 and a significant increase from baseline in old muscle at RL4 but not persisting to RL7 (Figure 2E). Importantly, COX‐associated oxylipins were present at markedly higher absolute concentrations than non‐enzymatic products, suggesting that COX‐derived mediators constitute a major contributor to the age‐associated oxylipin expansion during reloading (Figure 2D,E).

Among the LOX pathways, 5‐LOX showed only an age effect at HU, characterized by higher levels in young muscle, without a sustained increase during reloading (Figure 2F). 15‐LOX differed between ages only at the ambulatory baseline, again without a consistent increase (Figure 2G). In contrast, the 12‐LOX pathway demonstrated a selective age‐dependent increase at RL4, accompanied by a significant rise from baseline in old muscle at this timepoint, but this increase was not maintained at RL7 (Figure 2H). No statistically significant differences were detected in total CYP‐derived oxylipin concentrations across age or time (Figure 2I). Given these age‐related elevations in COX‐associated oxylipins during reloading, we next examined individual COX‐derived prostaglandins to characterize this response (Figure 2J). Analysis of individual prostaglandins revealed that aged muscle exhibited a broad increase in several COX‐derived lipid mediators during the recovery period following hindlimb unloading. Specifically, pro‐inflammatory prostaglandins, including PGE2, dhk PGE2, PGF2α, PGD2, 11b PGF2α, and TxB2, were significantly elevated in old muscle compared with young muscle during reloading (Figure 2J). In contrast, young muscle maintained relatively stable prostaglandin levels throughout recovery, suggesting that aging is associated with an exaggerated and sustained prostaglandin response following disuse.

We next examined the relative changes of individual oxylipin species across the disuse and reloading time course. Heat map visualization showed pronounced relative increases in several oxylipins in aged muscle, including those associated with the 15‐LOX, 12‐LOX, and CYP pathways. In contrast, young muscle displayed predominantly transient fluctuations across time points (Figure S3A). Collectively, these data indicate that skeletal muscle reloading in aging is accompanied by an exaggerated and prolonged expansion of oxylipin pools rather than the more acute and self‐resolving response observed in young muscle. This aged phenotype was further characterized by preferential expansion of omega‐6‐linked and COX‐associated lipid mediator pools (Figure 2B,D).

To determine whether these age‐associated lipid mediator patterns were conserved in humans, we next examined oxylipin remodeling in quadriceps muscle following unilateral leg immobilization and recovery. In brief, young and older, healthy male adults underwent a 2‐week period of single‐leg immobilization, followed by 7 days of recovery. Muscle biopsies were collected prior to single‐leg immobilization (Pre), right after 2 weeks of single‐leg immobilization (Post) and at 2 (RL2) and 7 (RL7) days of recovery (Figure 2K). Relative abundance of Pathway‐level distributions revealed relatively stable contributions of the COX and LOX pathways across time in young participants. Similar to our mouse analysis, oxylipins were grouped by fatty acid origin and enzymatic pathways (Figure 2L–U). Unlike the pronounced remodeling observed in aged mice, human skeletal muscle did not exhibit significant changes in overall oxylipin content derived from enzymatic pathways during recovery. Nevertheless, given the prominent COX signature identified in aged mice, we next examined whether individual COX‐derived prostaglandins displayed a similar response in humans (Figure 2V). Analysis of individual prostaglandin species revealed a comparable age‐associated increase during recovery, with PGE2 and PGF2α significantly elevated in older adults at RL7 compared with young adults. Additionally, dhk PGE2 approached statistical significance, while PGD2 and TxB2 showed similar upward trends in older adults following disuse. 11b PGF2α was not detected in human skeletal muscle samples (Figure 2V).

Analysis of individual oxylipin species further highlighted these differences. Young muscle exhibited early, transient increases in multiple mediators that partially resolved by RL7, whereas older muscle showed broader and more sustained accumulation of lipid mediators at RL7 (Figure S3B). Collectively, these findings indicate that skeletal muscle recovery in older humans is accompanied by amplified oxylipin remodeling that recapitulates the accumulation of lipid mediators observed in aged mice. Although responses occurred earlier and were higher in magnitude in the pre‐clinical model, both the pre‐clinical and clinical datasets converged on a preferential expansion of omega‐6‐derived and COX‐associated prostaglandin pathways.

### 3.3 COX‐Prostaglandin Signaling Links Senescence Burden and Impaired Muscle Recovery in Aged Mice

To determine whether altered lipid mediator remodeling during reloading was linked to cellular senescence and functional recovery, we next examined senescence and SASP burden and their relationships with COX‐derived oxylipins in young and old skeletal muscle.

Quantification of SA‐β‐Gal‐positive cells in the gastrocnemius revealed that aged mice exhibited a significant accumulation of senescent positive cells at RL4, which further increased at RL7, whereas young muscle remained unchanged across all time points. Within‐group comparisons confirmed unchanged levels of senescent cells in young mice, whereas old mice showed a progressive increase from baseline to RL7 (Figure 3A). Correlation analyses performed at RL7 showed no relationship between SA‐β‐Gal^+^ cells and muscle mass in young mice. In contrast, aged mice displayed a significant negative correlation, with higher senescence burden strongly associated with lower gastrocnemius mass (Figure 3B). These findings indicate that, in aged muscle, senescent cell accumulation is linked to impaired muscle mass recovery after disuse.

**FIGURE 3:**
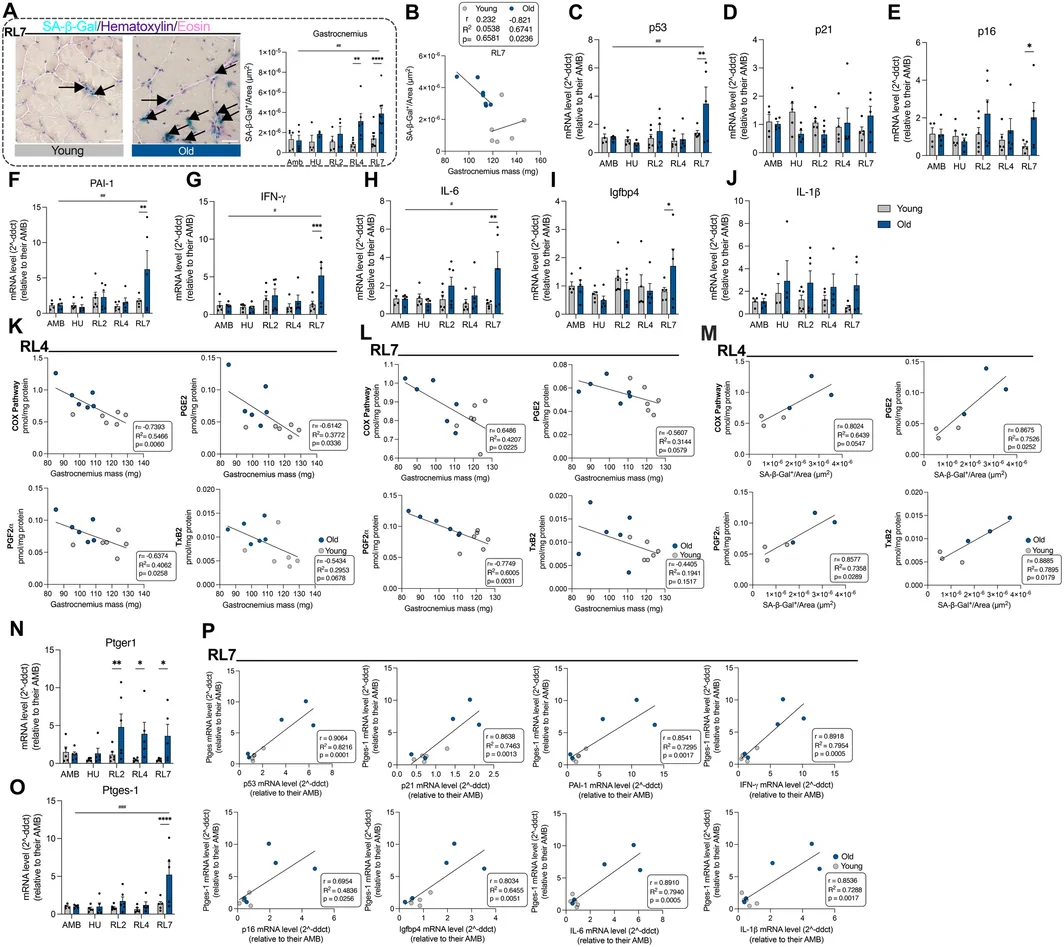
COX‐prostaglandin pathway activation correlates with senescence burden during impaired muscle recovery in aged mice. (A) Representative images of SA‐β‐Gal counterstained with H&E histology in gastrocnemius muscle from young and old mice at RL7. Quantitative analysis in gastrocnemius of young and old mice at AMB, HU, RL2, RL4, and RL7 (*n* = 3–10/group and time point). (B) Correlation analysis of SA‐β‐Gal^+^/Area (μm^2^) and gastrocnemius muscle mass (mg) in young and old mice at RL7 (*n* = 6–7). (C–E) mRNA level of Senescence markers (p53, p21, p16) by RT‐qPCR at AMB, HU, RL2, RL4, and RL7 (*n* = 4–6/group and time point). (F–J) mRNA level of SASP associated markers (PAI‐1, IFN‐γ, Igfbp4, IL‐6, IL‐1β) by RT‐qPCR at AMB, HU, RL2, RL4, and RL7 (*n* = 4–6/group and time point). (K) Correlation analysis of gastrocnemius mass and the COX pathway with major downstream oxylipins (PGE2, PGF2α, TxB2) at RL4. Pearson's correlation, with corresponding *p*‐values, *r*, and *R* ^2^ displayed on the graph (*n* = 6/group). (L) Correlation analysis of gastrocnemius mass and the COX pathway with major downstream oxylipins (PGE2, PGF2α, TxB2) at RL7. Pearson's correlation, with corresponding *p*‐values, *r*, and *R* ^2^ displayed on the graph (*n* = 6/group). (M) Correlation analysis of SA‐β‐Gal^+^/Area (μm^2^) and the COX pathway with major downstream oxylipins (PGE2, PGF2α, TxB2) at RL4. Pearson's correlation, with corresponding *p*‐values, *r* and *R* ^2^ displayed on the graph (*n* = 3/group and timepoint). (N) mRNA level of Ptger1 by RT‐qPCR at AMB, HU, RL2, RL4, and RL7 (*n* = 4–6/group and timepoint). (O) mRNA level of Ptges‐1 by RT‐qPCR at AMB, HU, RL2, RL4, and RL7 (*n* = 4–6/group and timepoint). (P) Correlation analysis of senescence (p53, p21, p16) and SASP associated markers (PAI‐1, IFN‐γ, Igfbp4, IL‐6, IL‐1β), mRNA level and Ptges mRNA level at RL7. Pearson's correlation, with corresponding *p*‐values, *r* and *R* ^2^ displayed on the graph (*n* = 5/group). Statistical analysis: All data presented as mean ± SEM. Statistical analyses were performed using two‐way ANOVA followed by Tukey's multiple‐comparisons test. *p < 0.05, **p < 0.01, ***p < 0.001, and ****p < 0.0001. (*) indicates a group difference at a specific time point. #*p* < 0.05, ##*p* < 0.01, ###*p* < 0.001, and ####*p* < 0.0001. (#) indicates a within group difference from their baseline time point. Correlations were evaluated using Pearson's correlation. Cell specific identification is indicated by a black arrow. Scale bar 30 μm.

Analysis of senescence markers and SASP transcripts revealed time‐ and age‐dependent changes that were most evident during late reloading (RL7; Figure 3C–J). Expression of p53 and p16 was significantly higher in old compared with young gastrocnemius muscle at RL7, whereas p21 remained unchanged across age groups and time points. Notably, p53 was significantly increased from baseline to RL7 within the old group (Figure 3C–E). Among SASP‐associated genes, PAI‐1, IFN‐γ, Igfbp4, and IL‐6 were preferentially elevated in aged muscle at RL7, with PAI‐1, IFN‐γ, and IL‐6 also showing significant increases from baseline to RL7 within the old group (Figure 3F–I). In contrast, IL‐1β remained unchanged across age groups and time points (Figure 3J). Collectively, these findings support the emergence of a senescence‐associated transcriptional program in aged muscle during recovery.

To evaluate whether COX pathway lipid mediator content is related to functional recovery, we performed correlation analyses between gastrocnemius mass and COX pathway activity at RL4 and RL7. Muscle mass was inversely associated with total COX‐linked oxylipin abundance, as well as with major downstream prostaglandins PGE2 and PGF2α, whereas the association with TxB2 did not reach statistical significance (Figure 3K,L).

To determine whether this relationship extended beyond the COX pathway, we examined additional enzymatic and non‐enzymatic lipid routes (Figure S4A,B). At RL4, non‐enzymatic oxidation products, 5‐LOX, 15‐LOX, and CYP‐derived metabolites showed significant inverse correlations with gastrocnemius mass, with these associations driven primarily by the old group. 12‐LOX demonstrated a similar trend. However, these correlations were no longer evident at RL7. These findings indicate that early reloading in old muscle is characterized by broader lipid remodeling, but the sustained relationship between lipid mediator activity and impaired recovery is most consistent within the COX pathway. Notably, these relationships were driven primarily in aged muscle, which clustered toward higher COX and prostaglandin levels and lower muscle mass, whereas young muscle remained tightly grouped with lower COX lipid mediator levels and higher gastrocnemius mass.

Correlation analyses demonstrated that COX pathway activity and downstream prostaglandins were positively associated with SA‐β‐Gal^+^ cell accumulation at RL4 (Figure 3M), further linking increased COX‐derived lipid mediators to cellular senescence.

Among other pathways examined, only 12‐LOX activity showed a significant positive correlation with SA‐β‐Gal^+^ cell abundance at RL4, whereas 5‐LOX, 15‐LOX, CYP, and non‐enzymatic products did not (Figure S4C). This suggests that although multiple lipid pathways are activated during early recovery, the strongest and most consistent correlation with senescence burden is with the COX pathway.

In addition, expression of Ptges‐1, which encodes microsomal prostaglandin E synthase‐1 (mPGES‐1), increased during reloading (Figure 3O). mPGES‐1 converts COX‐derived PGH_2_ into PGE_2_, positioning it as a key enzyme in the COX pathway. Expression of Ptger1, which encodes the EP1 G protein‐coupled receptor (GPCR) mediating downstream PGE2 signaling, also increased (Figure 3N). Together, these changes indicate activation of the PGE2 synthesis‐signaling axis during recovery. Notably, Ptges‐1 expression at RL7 positively correlated with senescence markers and SASP‐associated transcripts (Figure 3P). Together, these data link exaggerated omega‐6‐driven COX‐prostaglandin remodeling and senescence burden to impaired skeletal muscle recovery after disuse in aging.

### 3.4 Pharmacological Inhibition of COX‐Prostaglandin Signaling Improves Functional Recovery and Reduces Senescence Burden in Aged Muscle

To test whether heightened COX‐prostaglandin signaling contributes to impaired recovery in aged muscle, we subjected old (~25‐month‐old) mice to hindlimb unloading followed by 7 days of reloading and administered a COX‐inhibitor intraperitoneally throughout the reloading period (Figure 4A). Mice received saline, low‐dose, or high‐dose COX‐inhibitor. We first confirmed that the drug inhibited its intended target. Expression of Ptges‐1 and Ptger1 was significantly reduced in COX‐inhibitor‐treated mice compared with saline controls (Figure 4B). Low‐dose COX‐inhibitor significantly decreased both transcripts, whereas high‐dose treatment reduced Ptger1 and showed a trend toward reduced Ptges‐1 expression (Figure S5A). These findings confirm effective suppression of the PGE2 synthesis‐signaling axis during reloading.

**FIGURE 4:**
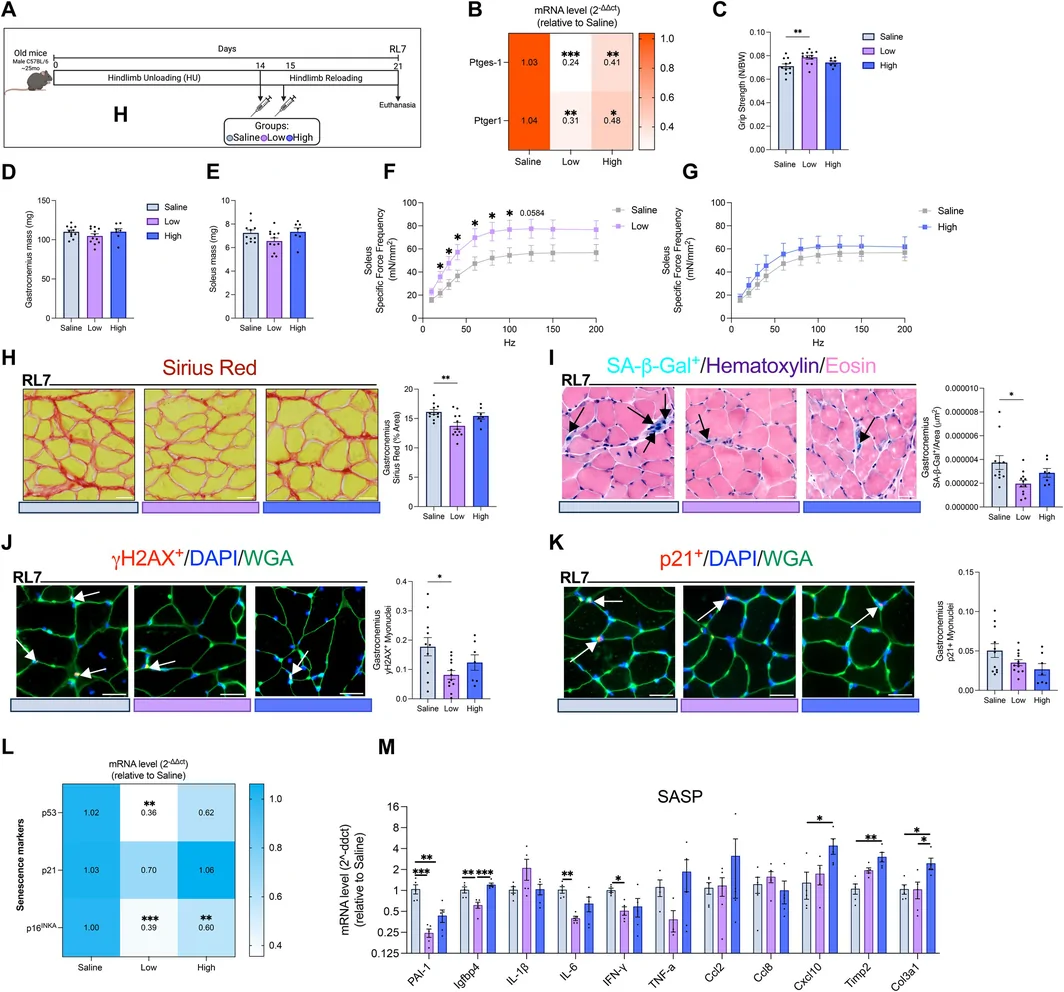
COX inhibition improves recovery and attenuates senescent cell accumulation and fibrosis during reloading in aged mice. (A) Experimental study design, Old (~25 month) male C57BL/6 mice underwent 14 days of hindlimb unloading followed by 7 days of reloading. At reload, mice were intraperitoneally (IP) injected with Saline, High‐ or low‐dose COX‐inhibitor (*n* = 7–12/group). (B) mRNA level of key prostaglandin‐signaling genes, Ptges‐1 and Ptger1 by RT‐qPCR at RL7 in gastrocnemius, shown as mean (*n* = 5/group). (C) Whole‐body grip strength normalized to body weight in Saline, High or Low‐dose COX‐inhibitor at RL7 (*n* = 7–12/group). (D) Gastrocnemius mass of Saline, High or Low‐dose COX‐inhibitor at RL7 (*n* = 7–12/group). (E) Soleus mass of Saline, High or Low‐dose COX‐inhibitor at RL7 (*n* = 7–12/group). (F) Ex vivo soleus specific force frequency (mN/mm^2^) from 10 to 200 Hz of Saline and Low dose (*n* = 11–12). (G) Ex vivo soleus specific force frequency (mN/mm^2^) from 10 to 200 Hz of Saline and High dose (*n* = 6–11). (H) Representative images and quantification of Sirius red staining in the gastrocnemius of Saline, High or Low‐dose COX‐inhibitor at RL7 (*n* = 7–12/group). (I) Representative images and quantification of SA‐β‐gal counterstained with H&E histology in gastrocnemius muscle Saline, High or Low‐dose COX‐inhibitor at RL7 (*n* = 7–12/group). (J) Representative images and quantification of γH2AX^+^ in gastrocnemius muscle, Saline, High or Low‐dose COX‐inhibitor at RL7 (*n* = 7–12/group). (K) Representative images and quantification of p21^+^ in gastrocnemius muscle Saline, High or Low‐dose COX‐inhibitor at RL7 (*n* = 7–12/group). (L) mRNA level of senescent markers, p53, p21, p16^INKA^by RT‐qPCR at RL7 in gastrocnemius, shown as mean (*n* = 5/group). (M) mRNA level of SASP markers, PAI‐1, Igfbp4, IL‐1β, IL‐6, IFN‐γ, TNF‐α, Ccl2, Ccl8, Cxcl10, Timp2, Col3a1 by RT‐qPCR at RL7 in gastrocnemius, (*n* = 5/group). Statistical analysis: All data presented as mean ± SEM. Data represent biologically independent muscle samples. *p < 0.05, **p < 0.01, and ***p < 0.001, as determined by one‐way ANOVA followed by Tukey's multiple comparisons test. Statistical analyses were performed with a two‐way ANOVA and Tukey's multiple comparison test F and G. Cell‐specific identification is indicated by a white or black arrow. Scale bar 30 μm.

Furthermore, whole‐body grip strength (normalized to body weight) was significantly higher in low‐dose‐treated mice compared with saline controls (Figure 4C). On the other hand, high‐dose treatment did not alter grip strength. Gastrocnemius and soleus mass were not significantly different among groups (Figure 4D,E), indicating that COX inhibition did not enhance functional recovery by increasing muscle mass.

Despite unchanged muscle mass, contractile capacity improved in the low‐dose group. Soleus specific force across stimulation frequencies was significantly higher in the low‐dose group (Figure 4F) with significant differences at mid‐to‐high frequencies. Similar improvements were observed in absolute soleus force at these frequencies (Figure S5G). In contrast, neither specific nor absolute force differed significantly between the high‐dose and saline groups (Figure 4G; Figure S5H). Importantly, the mean myofiber cross‐sectional area did not differ significantly across treatment groups in the gastrocnemius muscle, although analysis of the fiber‐size distribution revealed modest differences within specific size bins (Figure S5B,C). The proportion of centrally nucleated fibers and Pax7^+^ satellite cells was similar across groups (Figure S5D,E).

To further assess muscle quality, Sirius red staining was performed to evaluate collagen deposition. Low‐dose COX‐inhibitor significantly reduced fibrotic collagen content in the gastrocnemius muscles compared with saline controls (Figure 4H), whereas high‐dose treatment did not significantly affect fibrosis. These findings suggest that moderate COX inhibition reduces fibrotic accumulation during recovery of aged muscle.

Inhibition of COX‐prostaglandin signaling during reloading was accompanied by an evident decrease in senescence‐associated markers in aged muscle. SA‐β‐Gal‐positive area in gastrocnemius muscle was significantly lower in low‐dose‐treated mice compared with saline controls (Figure 4I), and γH2AX^+^ nuclei, a marker of DNA damage signaling, were similarly reduced (Figure 4J). In contrast, p21^+^ nuclei were not significantly altered (Figure 4K).

At the transcriptional level, low‐dose treatment significantly reduced p53 and p16 expression, whereas p21 mRNA remained unchanged (Figure 4L). High‐dose treatment also reduced p16 expression but had a weaker effect on p53 (Figure S5F).

Analysis of SASP‐associated transcripts further supported attenuation of the senescence program. Low‐dose COX‐inhibitor significantly reduced expression of PAI‐1, Igfbp4, IL‐6, and IFN‐γ compared with saline controls. In contrast, IL‐1β, TNF‐α, Ccl2, and Ccl8 were not significantly altered. High‐dose treatment selectively increased Cxcl10, Timp2, and Col3a1 expression, while also decreasing PAI‐1 (Figure 4M).

Collectively, these findings demonstrate that pharmacological inhibition of COX‐prostaglandin signaling during reloading improves functional recovery and reduces senescence burden in aged muscle. Together with our lipidomic/oxylipin data, these results provide functional validation that heightened and persistent COX‐driven lipid mediator activity contributes to senescence burden and impaired skeletal muscle recovery in aging.

### 3.5 Senescent Macrophages Promote COX‐2‐Dependent PGE2 Production and Induce Myotube Atrophy via Paracrine Signaling

To better understand the cellular mechanisms underlying the COX‐prostaglandin‐driven senescence, we developed an in vitro model of doxorubicin‐induced senescence using C2C12 myoblasts, RAW 264.7 macrophages, and NOR‐10 fibroblasts. These cell types play key roles in skeletal muscle recovery, extracellular matrix remodeling, and inflammatory signaling, processes that are disrupted with aging and susceptible to senescence. (Figure 5A). As expected, doxorubicin treatment caused a noticeable increase in senescence across all cell types, as shown by SA‐β‐Gal staining (Figure 5B). We then determined whether this senescent state was linked to changes in downstream COX‐2 lipids in the secretome/SASP. Interestingly, the effect on PGE2 was highly dependent on cell type (Figure 5C). In C2C12 myoblasts, PGE2 levels decreased following doxorubicin treatment, while RAW 264.7 macrophages showed a significant increase. In contrast, NOR‐10 fibroblasts did not show any noticeable change. Together, these data suggest that, among the cell types tested, macrophages are a major source of increased PGE2 production under senescent conditions.

**FIGURE 5:**
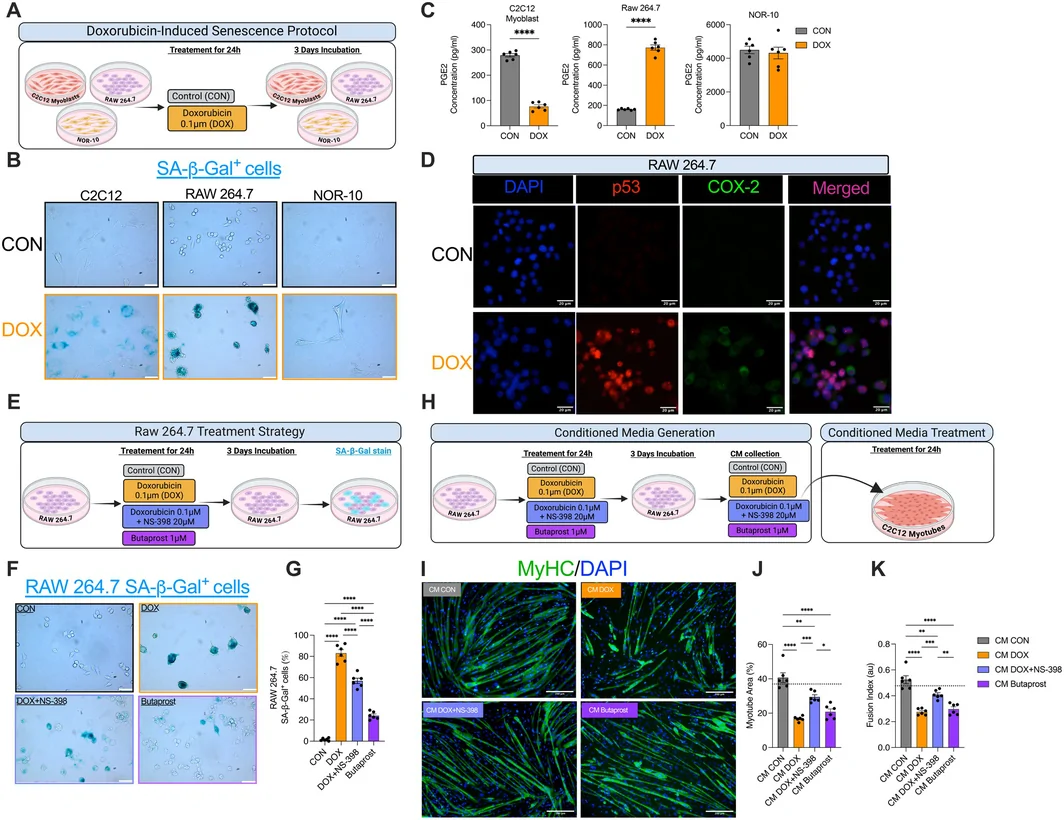
Senescent macrophages drive COX‐2‐dependent PGE2 production and induce myotube atrophy via secreted factors. (A) Experimental design of the doxorubicin (DOX)‐induced senescence protocol in C2C12 myoblasts, RAW 264.7 macrophages, and NOR‐10 fibroblasts. Cells were treated with DOX (0.1 μM, 24 h) followed by a 3‐day incubation. (B) Representative images of senescence‐associated β‐galactosidase (SA‐β‐Gal) staining in control (CON) and DOX‐treated C2C12, RAW 264.7, and NOR‐10 cells. (C) Prostaglandin E2 (PGE2) concentration measured by ELISA in culture media from CON and DOX‐treated C2C12, RAW 264.7, and NOR‐10 cells. (D) Representative immunofluorescence images of RAW 264.7 cells stained for DAPI (nuclei, blue), p53 (red), and cyclooxygenase‐2 (COX‐2, green) under CON and DOX conditions. (E) Experimental design for RAW 264.7 treatment conditions including CON, DOX, DOX + NS‐398 (COX‐2 inhibitor), and Butaprost (EP2 agonist). (F) Representative SA‐β‐Gal staining in RAW 264.7 cells across all treatment groups. (G) Quantification of SA‐β‐Gal‐positive RAW 264.7 cells (%). (H) Experimental design of conditioned media (CM) generation from RAW 264.7 cells treated with CON, DOX, DOX + NS‐398 (COX‐2 inhibitor), and Butaprost (EP2 agonist), followed by treatment of differentiated C2C12 myotubes with CM from each condition. (I) Representative immunofluorescence images of myotubes stained for MyHC (green) and DAPI (blue) following treatment with CM from each condition. (J) Quantification of myotube area (%). The dotted line represents myotubes differentiated under standard differentiation media (DM) conditions. (K) Quantification of fusion index (arbitrary units). The dotted line represents myotubes differentiated under standard differentiation media (DM) conditions. Statistical analysis: All data presented as mean ± SEM. Data represent biologically independent experiments (*n* = 6 per group). *p < 0.05, **p < 0.01 and ***p < 0.001, as determined by one‐way ANOVA followed by Tukey's multiple comparisons test (G, J, K). For PGE2 Elisa measurements, statistical significance was assessed using an unpaired two‐tailed *t*‐test. White scale bars: SA‐β‐Gal stain, 10 μm; immunofluorescence (DAPI/p53/COX‐2), 20 μm; MyHC/DAPI images, 200 μm.

Given this, we focused our subsequent analyses on RAW 264.7 macrophages. Doxorubicin treatment induced p53 and COX‐2 staining by immunofluorescence, whereas little to no signal was observed under control conditions (Figure 5D), further connecting COX‐2‐derived lipid signaling to cellular senescence. To test whether this pathway plays a functional role in senescence, we next pharmacologically modulated COX‐2 activity. Doxorubicin significantly increased the proportion of SA‐β‐Gal‐positive cells, while inhibition of COX‐2 with NS‐398 during doxorubicin treatment attenuated this response. In contrast, activating prostaglandin signaling with Butaprost (an EP2 prostaglandin receptor agonist) increased the proportion of senescent cells relative to control, although to a lesser extent than doxorubicin treatment (Figure 5F,G). These results suggest that activating PGE2 signaling alone is enough to induce a senescent phenotype, supporting a role for COX‐2‐dependent prostaglandin signaling in reinforcing cellular senescence in macrophages.

We then investigated whether factors secreted by these senescent macrophages with elevated PGE2 levels (Figure 5C) could influence muscle cells. CM from each treatment group were applied to differentiated C2C12 myotubes (Figure 5H). CM from doxorubicin‐treated macrophages reduced myotube area compared to control CM, indicating myotube atrophy (Figure 5J). This effect was partially mitigated when myotubes were treated with CM from DOX + NS‐398 treated macrophages, although myotube area did not completely return to control levels. In contrast, CM from Butaprost treated macrophages caused a similar reduction in myotube size as seen with CM from DOX. A similar response was observed for the fusion index (Figure 5K). CM from DOX‐treated macrophages decreased fusion capacity, while CM from DOX + NS‐398 treated macrophages partially restored it. CM from Butaprost‐treated macrophages mirrored the CM from DOX and remained lower than control.

Taken together, these findings demonstrate that senescent‐induced macrophages increase COX‐2‐dependent PGE2 production and, through their secretome, hinder myotube size and fusion. This reveals a mechanism that connects a cellular source to increased prostaglandin signaling and impaired muscle recovery.

## 4 Discussion

In this study, through lipidomics, oxylipin profiling, senescence analysis, and pharmacologic intervention, we identify heightened and sustained omega‐6‐derived COX signaling as a central feature of aged muscle during reloading after a period of disuse. We show that aging is associated with altered PC composition and increased LPC accumulation during recovery. This lipid remodeling phenotype is associated with increased senescence burden and SASP expression. Moderate COX inhibition improves functional recovery and reduces senescence and fibrosis, despite no significant increase in muscle mass. Importantly, similar age‐associated oxylipin remodeling patterns were observed in older adults following limb immobilization, supporting the translational relevance of these findings. Together, these data position prolonged COX‐prostaglandin activity as a mechanistic link between lipid remodeling, senescence burden, and impaired skeletal muscle recovery in aging.

A key upstream feature of the aged recovery response was altered membrane phospholipid composition. Membrane phospholipids are not only structural components of the cell but also major reservoirs for bioactive lipid mediator precursors, including esterified AA (Giannakis et al. 2019). Upon cellular stress or inflammatory activation, cytosolic cPLA2 cleaves off and releases these fatty acids for downstream oxylipin production (Czyzowska et al. 2023; Pharaoh et al. 2020). Sarcopenic muscle in older adults is characterized by elevated levels of major phospholipids, including PC, which is associated with reduced muscle mass and function (Hinkley et al. 2020). Consistent with this, young mice's muscle exhibited stable PC content and transient changes in lipid mediators. While aged mice's muscle showed sustained PC enrichment, increased LPC, and preferential mobilization of AA during reloading. This pattern suggests enhanced phospholipid turnover and selective availability of omega‐6 substrates rather than a global release of PUFAs. Given that PC represents a major reservoir of esterified AA, this remodeling likely primes aged muscle for amplified downstream prostaglandin production. Notably, omega‐3‐derived substrates did not show comparable sustained expansion, indicating that aging skews recovery‐associated lipid remodeling toward omega‐6‐dominant signaling. This shift may be driven by increased mobilization of AA from phospholipid pools, as previously shown by increased cPLA2 activity and COX‐2 levels in aged gastrocnemius muscle (Czyzowska et al. 2023). This is further reinforced by the intrinsic substrate preference of cPLA2 for AA, which promotes selective omega‐6 fatty acid release from membrane phospholipids (Murakami et al. 2020; Hayashi et al. 2021; Mouchlis et al. 2018).

Consistent with this substrate bias, targeted oxylipin profiling revealed that skeletal muscle reloading in aged mice is characterized by a pronounced and persistent expansion of omega‐6‐derived lipid mediators, particularly those associated with the COX pathway. In young muscle, oxylipin responses were largely transient. In contrast, aged muscle demonstrated early and sustained elevations in total oxylipins, with COX‐derived prostaglandins emerging as the dominant and most persistent contributors. This pattern is notable because lipid mediator responses during muscle repair are typically transient and resolve over time, as observed following acute resistance exercise in humans (Vella et al. 2019). Although other lipid pathways, including 12‐LOX and non‐enzymatic oxidation products, showed transient associations with muscle mass during early reloading, the sustained relationship between lipid mediator burden and impaired recovery was most consistent within the COX pathway. These findings suggest that aging shifts the balance from a coordinated, resolving lipid response toward prolonged and heightened COX‐prostaglandin‐driven signaling. The contributions of LOX and CYP pathways to impaired muscle recovery warrant further investigation, particularly given evidence implicating 12/15‐LOX‐associated oxylipins and lipid hydroperoxides in denervation‐induced muscle atrophy, a process relevant to sarcopenia (Brown et al. 2022).

Importantly, heightened COX activity was tightly linked to the emergence of cellular senescence during recovery. Senescent cell burden in aged muscle increased progressively during reloading (Bourrant et al. 2026) and correlated negatively with muscle mass and positively with COX‐derived prostaglandins and pathway activity. Expression of Ptges‐1 and Ptger1, encoding key components of the PGE2 synthesis‐signaling axis, increased during reloading and correlated with senescence and SASP‐associated transcripts. These findings support a model in which persistent COX‐PGE2 signaling reinforces senescence‐associated programs in aged muscle. In addition, lipid mediators have been implicated in regulating senescence‐associated signaling and immune surveillance in both cell culture and mouse models, supporting the idea that prostaglandin excess may contribute to maladaptive remodeling rather than simply being a byproduct of tissue damage (Wiley et al. 2021; Srour et al. 2024; Goncalves et al. 2021). Consistent with this, PGE2 has been shown to directly promote and maintain the senescent phenotype (Srour et al. 2024; Goncalves et al. 2021; Martien et al. 2013; Han et al. 2004; Chou et al. 2014). Importantly, PGE2 supports satellite cell proliferation and skeletal muscle regeneration after acute muscle injury (Ho et al. 2017). PGE2 also has beneficial effects in aging skeletal muscle. In aged mice, inhibition of the PGE2‐degrading enzyme 15‐PGDH increases muscle mass and strength (Palla et al. 2021), while restoration of PGE2–EP4 signaling improves muscle stem‐cell function and regeneration following injury (Wang et al. 2025). Differences in cellular context may help explain these contrasting outcomes, as studies of satellite cell‐mediated regeneration did not focus on macrophages as a potential source of PGE2 secretion, which our findings highlight. This distinction may be particularly relevant during muscle regrowth after disuse, in which satellite cells are not necessary (Jackson et al. 2012) yet muscle regrowth is impaired by macrophage depletion (Tidball and Wehling‐Henricks 2007). In this framework, PGE2 signaling is tightly regulated, and the context, dose, and time‐dependent dynamics are critical determinants of its function. While transient, localized PGE2 signaling promotes regeneration, the current study supports that sustained or excessive PGE2 signaling in aged muscle during recovery may instead reinforce senescence‐associated pathways and impair effective skeletal muscle recovery. These findings highlight the importance of the physiological and cellular context when interpreting the effects of PGE2.

We also show that moderate pharmacologic suppression of COX signaling during reloading improved recovery in aged mice. COX‐inhibition improved grip strength and intrinsic contractile force production without increasing muscle mass, indicating improved muscle quality rather than hypertrophy effects. This functional improvement was accompanied by reduced fibrotic collagen deposition and attenuation of senescence markers and SASP‐associated transcripts. These findings provide mechanistic support for the hypothesis that heightened COX‐driven lipid mediator activity contributes to impaired recovery and increased senescence burden in aging muscle. At the same time, the dose‐dependent effects of COX‐inhibition observed here highlight the complexity of prostaglandin biology in skeletal muscle. While prostaglandins can support effective acute repair in some regenerative settings, their effects are highly dependent on timing, receptor usage, COX‐1 or COX‐2 preference, and tissue context (Ho et al. 2017; Wang et al. 2025). High‐dose COX‐inhibitor failed to improve function and altered fibrotic and inflammatory transcripts, underscoring that complete COX suppression may disrupt muscle remodeling. These results may help reconcile previously divergent findings regarding NSAID effects on muscle regeneration and emphasize the importance of dose, timing, and physiological context when modulating COX‐prostaglandin pathways in aging tissue (Mallinson et al. 2026, 2025; Mandic et al. 2026; Mackey et al. 2007; Lilja et al. 2018). Notably, chronic COX inhibition during resistance training in older adults has been shown to enhance muscle mass and strength, potentially by suppressing PGE2‐driven pathways linked to muscle atrophy (Trappe et al. 2013). This suggests that its benefits may be context‐dependent and most pronounced when COX‐derived signaling is sustained or elevated, as observed in aging, potentially through suppression of PGE2‐driven pathways linked to muscle atrophy (Liu et al. 2016; Trappe and Liu 2013).

Importantly, age‐associated oxylipin remodeling patterns observed in mice were recapitulated in older adults undergoing limb immobilization and recovery. Although human responses exhibited slower kinetics and lower absolute magnitudes than the preclinical model, older participants showed a preferential expansion of omega‐6‐derived and COX‐associated pathways during recovery, whereas young adults showed a more transient response. We acknowledge that the timing of the oxylipin muscle response from mouse to human was not identical. Differences in oxylipin kinetics may have occurred due to the differences in the disuse protocols (hindlimb unloading vs. unilateral limb immobilization), muscles sampled (gastrocnemius vs. vastus lateralis biopsy), and the rate of muscle recovery between species. Despite these differences, the convergence of preclinical and clinical datasets strengthens the translational significance of persistent prostaglandin signaling as a hallmark of impaired recovery in aging.

Our data also identify macrophages as a potential cell source contributing to this mechanism. COX‐2 expression has been localized to CD68^+^ macrophages in regenerating human skeletal muscle (Saclier et al. 2013), indicating that these cells contribute to prostaglandin production during repair. This signaling may become dysregulated with aging during reloading following disuse, as observed in our dataset. While macrophages are central regulators of skeletal muscle repair (Tidball and Wehling‐Henricks 2007; Liu et al. 2017; Fix, Ekiz, et al. 2021), our in vitro findings support that under cellular senescent conditions, these cells become a major source of COX‐2‐dependent PGE2 production. Consistent with this, senescent macrophages exhibit increased COX‐2 expression and PGE2 production with aging (Hayek et al. 1997; Chen et al. 2013). Notably, prior work has shown that COX‐2 inhibition reduces doxorubicin‐induced p53 accumulation by suppressing ROS‐mediated JNK activation (Kim and Shim 2016), supporting a link between COX‐2 activity and senescence‐associated pathways. Importantly, CM from senescent macrophages impaired myotube size and fusion, effects that were partially rescued by COX‐2‐specific inhibition (NS‐398), indicating a paracrine mechanism. In parallel, pharmacological activation of PGE2 signaling through the EP2 agonist, butaprost, was sufficient to induce a senescent phenotype, supporting a direct role for prostaglandin signaling in reinforcing senescence. As previously described, senescent cells promote macrophages to adopt an anti‐inflammatory phenotype (Zhang et al. 2024), which we have noted previously to accumulate in skeletal muscle of aged mice following disuse (Bourrant et al. 2026). PGE2 produced by senescent cells has been shown to drive this phenotype (Mazzoni et al. 2019). Together, these results suggest that prostaglandin signaling not only reflects inflammatory activity but actively drives senescence, shapes macrophage function, and potentially has direct impacts on muscle. Future in vivo gain and loss of function studies are needed to identify which cell types drive prostaglandin production and contribute to cellular senescence in aging muscle.

We acknowledge that the findings from this study are limited to male mice and humans. Further work is needed to determine whether females show similar responses during recovery and following COX inhibition, especially given that sex differences in skeletal muscle aging have been reported in humans (de Jong et al. 2023) and that women with sarcopenia are more prone to severe disability and increased mortality risk (Batsis et al. 2014).

In summary, our findings support a model in which aging skews membrane phospholipid remodeling toward enhanced omega‐6 substrate availability and sustained COX‐prostaglandin signaling during skeletal muscle reloading in both mice and humans. This persistent and heightened response of lipid mediators is associated with senescence accumulation, fibrotic remodeling, and impaired functional recovery. Moderate suppression of COX activity mitigates these maladaptive features, highlighting a potential therapeutic window for selectively modulating prostaglandin signaling in aging muscle.

## Author Contributions

Conceptualization: P.‐E.B. and M.J.D. Methodology: P.‐E.B., E.M.Y., R.J.C., Z.J.F., K.F., and M.J.D. Investigation: P.‐E.B., Z.J.F., E.M.Y., R.J.C., P.J.F., and A.S.Y. Visualization: P.‐E.B., E.M.Y., and M.J.D. Validation: P.‐E.B. and M.J.D. Formal analysis: P.‐E.B., Z.J.F., A.S.Y., and M.J.D. Funding acquisition: P.‐E.B., Z.J.F., E.M.Y., and M.J.D. Project administration: P.‐E.B. and M.J.D. Supervision: P.‐E.B. and M.J.D. Writing – original draft: P.‐E.B. and M.J.D. Writing – review and editing: P.‐E.B., E.M.Y., Z.J.F., R.J.C., C.M.S., K.F., and M.J.D.

## Funding

Funding for these experiments was provided by National Institutes of Health R01AG076075 (to M.J.D.), American Heart Association 25PRE1362056 (to P.‐E.B.), American Heart Association 26POST1548675 (to Z.J.F.), and National Institutes of Health 5T32DK091317 (to Z.J.F. and E.M.Y.).

## Conflicts of Interest

The authors declare no conflicts of interest.

## Supporting information


**Figure S1:** Distinct lipidomic signatures and their correlation to impaired muscle recovery in young and old mice. (A–D) Soleus, plantaris, tibialis anterior (TA), and extensor digitorum longus (EDL) muscle mass in young and old mice at AMB, HU, RL2, RL4, and RL7. Data represent biologically independent muscle samples (*n* = 6/group and time point). (E) Correlation analysis of whole‐body grip strength and the major hindlimb muscles (soleus, plantaris, TA, and EDL) at RL7. Pearson's correlation, with corresponding *p*‐values, *r* and *R* ^2^ displayed on the graph (*n* = 6/group and timepoint). (F) Principal component analysis (PCA) score plots of lipidomic profiles in gastrocnemius of young and old mice at AMB, HU, RL2, RL4, and RL7(*n* = 6/group and time point). (G) Correlation analysis of gastrocnemius muscle mass (mg) and total abundance of each lipid class: Cardiolipin (CL), Lysophosphatidylcholine (LPC), Lysophosphatidylethanolamine (LPE), Phosphatidylcholine (PC), Phosphatidylethanolamine (PE), Phosphatidylinositol (PI), Phosphatidylglycerol (PG), Phosphatidylserine (PS), Sphingomyelin (SM), Triacylglycerol (TAG), and Ceramide (CER), in young and old mice at RL7. Pearson's correlation, with corresponding *p*‐values, *r* and *R* ^2^ displayed on the graph (*n* = 6/group). Statistical analysis: All data presented as mean ± SEM. Statistical analyses were performed using two‐way ANOVA followed by Tukey's multiple‐comparisons test. *p < 0.05, **p < 0.01 (*) indicates a group difference at a specific time point. #*p* < 0.05, ##*p* < 0.01, ###*p* < 0.001, ####*p* < 0.0001 (#) indicates a within group difference from their baseline time point. Correlations were evaluated using Pearson's correlation.

**Figure S2:** OXPHOS and oxidative stress dynamics during disuse and recovery in young and old mice. (A–C) Western blot analysis of OXPHOS subunits in the gastrocnemius muscle of young and old mice at AMB, HU, RL2, RL4, and RL7. Ponceau staining was used as a loading control (*n* = 6/group and timepoint). (D) Malondialdehyde (MDA) levels were measured in the gastrocnemius of young and old mice at AMB, HU, RL2, RL4, and RL7 (*n* = 6/group and timepoint). Statistical analysis: All data presented as mean ± SEM. Statistical analyses were performed using two‐way ANOVA followed by Tukey's multiple‐comparisons test. *p < 0.05, **p < 0.01 (*) indicates a group difference at a specific time point.

**Figure S3:** Heat map of skeletal muscle oxylipin changes following immobilization and recovery in mouse and human models. (A) Heat map representing the average percentage change from AMB in oxylipin concentration detected in the mice gastrocnemius muscle at AMB, HU RL2, RL4, and RL7. Oxylipins are expressed relative to their combined (*n* = 6/groups) average baseline levels (% change). Increases in oxylipin levels are indicated by red, decreases by blue, detectable but unchanged levels in white, and above +100% range in dark red (*n* = 6/group and timepoint). (B) Heat map representing the average percentage change from Pre in oxylipins concentrations detected in human quadriceps muscle at Pre, Post, RL2, and RL7. Oxylipins are expressed relative to their combined (*n* = 5–6/groups) average baseline levels (% change). Increases in oxylipin levels are indicated by red, decreases by blue, detectable but unchanged levels in white, and above +100% range in dark red (*n* = 5–6/group and time point).

**Figure S4:** Additional enzymatic and non‐enzymatic oxylipin pathways correlate with impaired muscle recovery and increased senescence. (A) Correlation analysis of gastrocnemius mass with non‐enzymatic and major enzymatic pathways (5‐LOX, 12‐LOX, 15‐LOX, and CYP) at RL4. Pearson correlations with corresponding *p*‐values, *r*, and *R* ^2^ are shown on each graph (*n* = 6/group). (B) Correlation analysis of gastrocnemius mass with non‐enzymatic and major enzymatic pathways (5‐LOX, 12‐LOX, 15‐LOX, and CYP) at RL7. Pearson correlations with corresponding *p*‐values, *r*, and *R* ^2^ are shown on each graph (*n* = 6/group). (C) Correlation analysis of SA‐β‐Gal^+^/Area (μm^2^) with non‐enzymatic and major enzymatic pathways (5‐LOX, 12‐LOX, 15‐LOX, and CYP) at RL4. Pearson correlations with corresponding *p*‐values, *r*, and *R* ^2^ are shown on each graph (*n* = 3/group). Statistical analysis: Correlations were evaluated using Pearson's correlation.

**Figure S5:** COX inhibition effects on prostaglandin signaling, muscle regeneration, and senescence during reloading. (A) mRNA level of key prostaglandin‐signaling genes, *Ptges‐1* and *Ptger1* by RT‐qPCR at RL7 in gastrocnemius, (*n* = 5/group). (B) Representative images and quantification of gastrocnemius myofibrillar mean CSA (*n* = 6–12). (C) Gastrocnemius fiber size distribution (*n* = 6–12). (D) Representative images and quantification of gastrocnemius PAX7^+^ cells (*n* = 6–12). (E) Representative images and quantification of centrally nucleated fibers (CNF) in the gastrocnemius (*n* = 6–12). (F) mRNA level of senescent markers, *p53*, p21, p16^INKA^by RT‐qPCR at RL7 in gastrocnemius, Data represent biologically independent muscle samples (*n* = 5/group). (G) Ex vivo soleus absolute force frequency (mN) from 10 to 200 Hz of Saline and Low dose (*n* = 11–12). (H) Ex vivo soleus absolute force frequency (mN) from 10 to 200 Hz of Saline and High dose (*n* = 6–11). Statistical analysis: All data presented as mean ± SEM Data represent biologically independent muscle samples. *p < 0.05, **p < 0.01 and ***p < 0.001, as determined by one‐way ANOVA followed by Tukey's multiple comparisons test. (A, B, D, E, F) Statistical analyses were performed with a two‐way ANOVA and Tukey's multiple comparison test C, G, H. Cell‐specific identification is indicated by a white arrow. Scale bar 30 μm.

## Data Availability

The data that support the findings of this study are available on request from the corresponding author. The data are not publicly available due to privacy or ethical restrictions.
