# Supplementary material for: Excessive COX‐Prostaglandin Signaling Promotes Cellular Senescence and Impaired Skeletal Muscle Recovery in Aging
Source: Aging Cell. 2026 Nov 13;25(11):e70843. doi: 10.1111/acel.70843 (PMC13768205; doi:10.1111/acel.70843)
Supplement: Supplementary file 1 [file ACEL-25-e70843-s001.pdf]

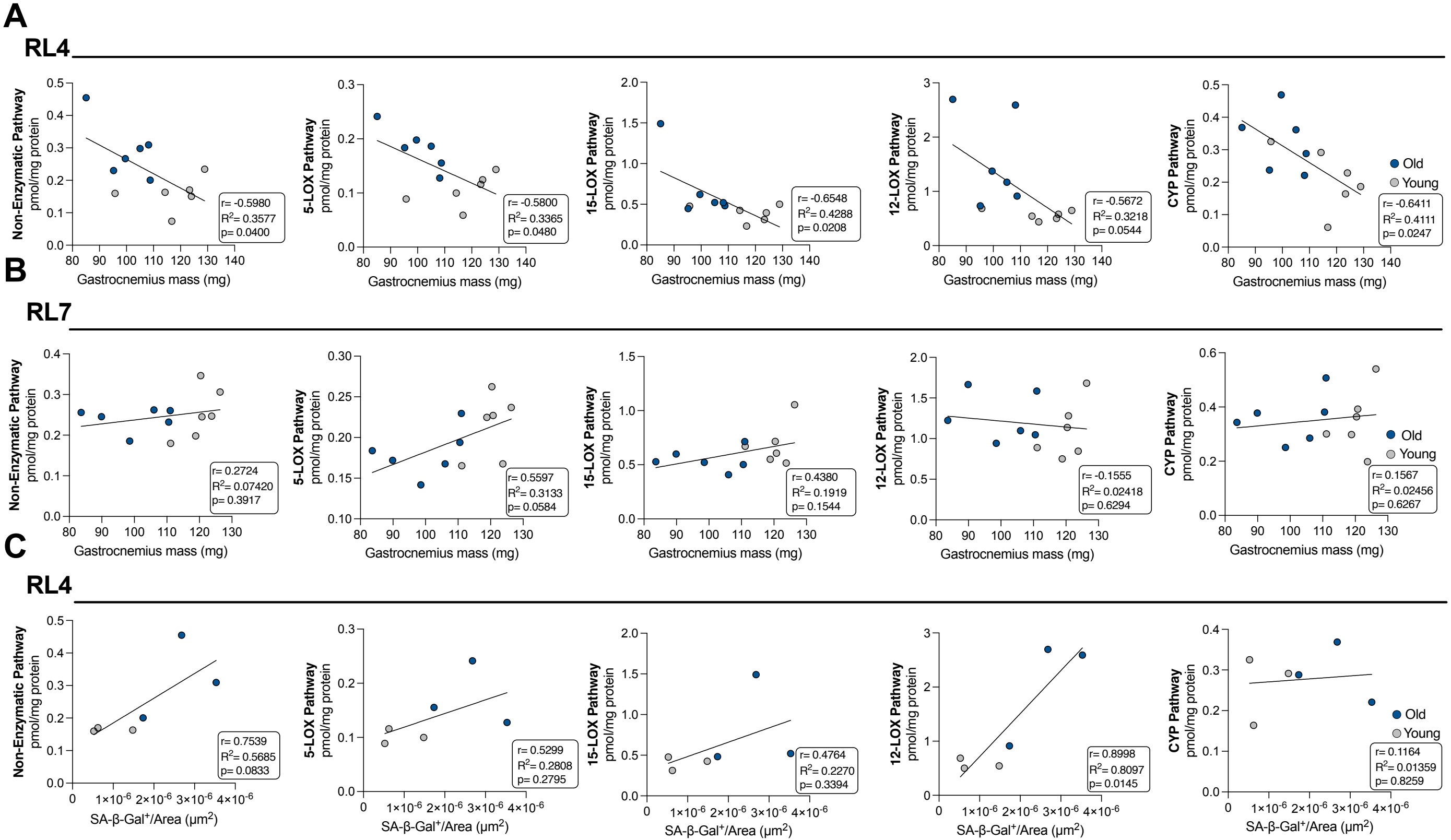

A
RL4
Non-Enzymatic Pathway pmol/mg protein
Gastrocnemius mass (mg)
r= -0.5980
R^2= 0.3577
p= 0.0400
5-LOX Pathway pmol/mg protein
r= -0.5800
R^2= 0.3365
p= 0.0480
15-LOX Pathway pmol/mg protein
r= -0.6548
R^2= 0.4288
p= 0.0208
12-LOX Pathway pmol/mg protein
r= -0.5672
R^2= 0.3218
p= 0.0544
CYP Pathway pmol/mg protein
r= -0.6411
R^2= 0.4111
p= 0.0247
Old
Young
B
RL7
Non-Enzymatic Pathway pmol/mg protein
Gastrocnemius mass (mg)
r= 0.2724
R^2= 0.07420
p= 0.3917
5-LOX Pathway pmol/mg protein
r= 0.5597
R^2= 0.3133
p= 0.0584
15-LOX Pathway pmol/mg protein
r= 0.4380
R^2= 0.1919
p= 0.1544
12-LOX Pathway pmol/mg protein
r= -0.1555
R^2= 0.02418
p= 0.6294
CYP Pathway pmol/mg protein
r= 0.1567
R^2= 0.02456
p= 0.6267
Old
Young
C
RL4
Non-Enzymatic Pathway pmol/mg protein
SA-β-Gal⁺/Area (μm²)
r= 0.7539
R^2= 0.5685
p= 0.0833
5-LOX Pathway pmol/mg protein
r= 0.5299
R^2= 0.2808
p= 0.2795
15-LOX Pathway pmol/mg protein
r= 0.4764
R^2= 0.2270
p= 0.3394
12-LOX Pathway pmol/mg protein
r= 0.8998
R^2= 0.8097
p= 0.0145
CYP Pathway pmol/mg protein
r= 0.1164
R^2= 0.01359
p= 0.8259
Old
Young
