# Supplementary material for: Excessive COX‐Prostaglandin Signaling Promotes Cellular Senescence and Impaired Skeletal Muscle Recovery in Aging
Source: Aging Cell. 2026 Nov 13;25(11):e70843. doi: 10.1111/acel.70843 (PMC13768205; doi:10.1111/acel.70843)
Supplement: Supplementary file 2 [file ACEL-25-e70843-s002.pdf]

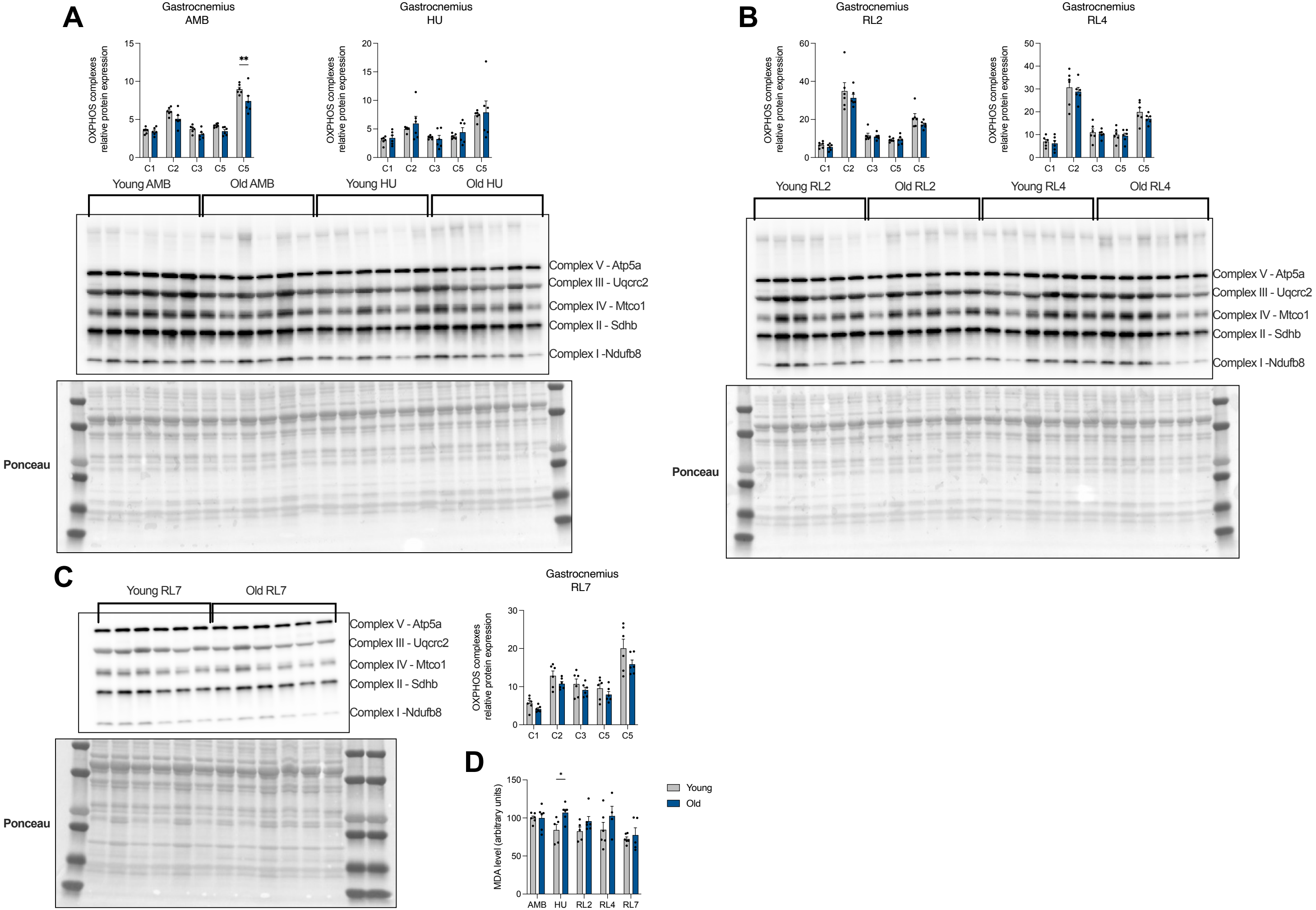

A
Gastrocnemius AMB
Gastrocnemius HU
OXPHOS complexes relative protein expression
C1 C2 C3 C5 C5
**
Young AMB
Old AMB
Young HU
Old HU
Complex V - Atp5a
Complex III - Uqcrc2
Complex IV - Mtco1
Complex II - Sdhb
Complex I -Ndufb8
Ponceau
B
Gastrocnemius RL2
Gastrocnemius RL4
Young RL2
Old RL2
Young RL4
Old RL4
C
Young RL7
Old RL7
Gastrocnemius RL7
D
MDA level (arbitrary units)
AMB HU RL2 RL4 RL7
*
Young
Old
