# Supplementary material for: Excessive COX‐Prostaglandin Signaling Promotes Cellular Senescence and Impaired Skeletal Muscle Recovery in Aging
Source: Aging Cell. 2026 Nov 13;25(11):e70843. doi: 10.1111/acel.70843 (PMC13768205; doi:10.1111/acel.70843)
Supplement: Supplementary file 3 [file ACEL-25-e70843-s003.pdf]

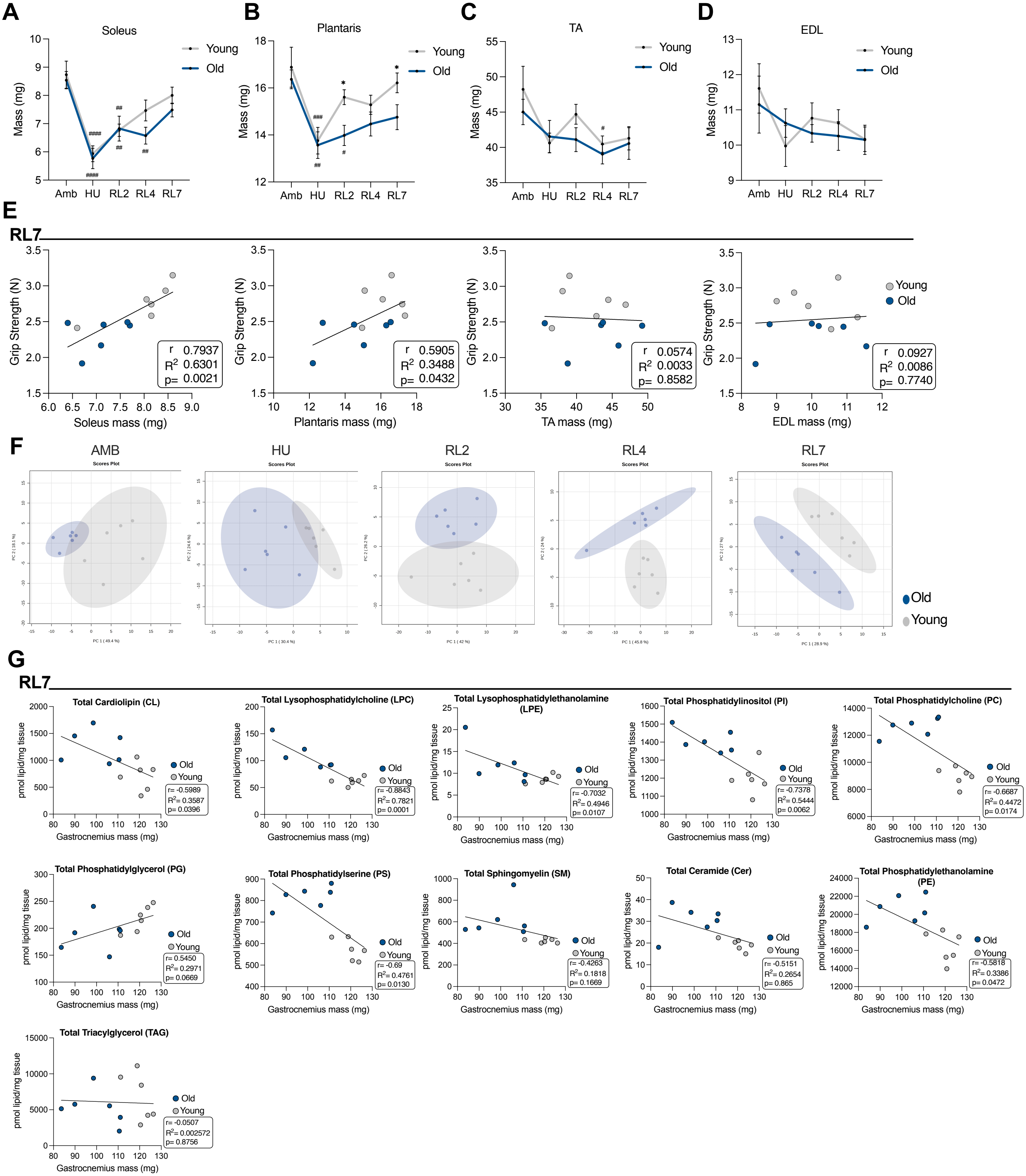
A
Soleus
Young
Old
Mass (mg)
Amb HU RL2 RL4 RL7
B
Plantaris
C
TA
D
EDL
E
RL7
Grip Strength (N)
Soleus mass (mg)
r 0.7937
R^2 0.6301
p= 0.0021
Plantaris mass (mg)
r 0.5905
R^2 0.3488
p= 0.0432
TA mass (mg)
r 0.0574
R^2 0.0033
p= 0.8582
EDL mass (mg)
r 0.0927
R^2 0.0086
p= 0.7740
F
AMB
HU
RL2
RL4
RL7
Scores Plot
Old
Young
G
RL7
Total Cardiolipin (CL)
pmol lipid/mg tissue
Gastrocnemius mass (mg)
r= -0.5989
R^2= 0.3587
p= 0.0396
Total Lysophosphatidylcholine (LPC)
r= -0.8843
R^2= 0.7821
p= 0.0001
Total Lysophosphatidylethanolamine (LPE)
r= -0.7032
R^2= 0.4946
p= 0.0107
Total Phosphatidylinositol (PI)
r= -0.7378
R^2= 0.5444
p= 0.0062
Total Phosphatidylcholine (PC)
r= -0.6687
R^2= 0.4472
p= 0.0174
Total Phosphatidylglycerol (PG)
r= 0.5450
R^2= 0.2971
p= 0.0669
Total Phosphatidylserine (PS)
r= -0.69
R^2= 0.4761
p= 0.0130
Total Sphingomyelin (SM)
r= -0.4263
R^2= 0.1818
p= 0.1669
Total Ceramide (Cer)
r= -0.5151
R^2= 0.2654
p= 0.865
Total Phosphatidylethanolamine (PE)
r= -0.5818
R^2= 0.3386
p= 0.0472
Total Triacylglycerol (TAG)
r= -0.0507
R^2= 0.002572
p= 0.8756
