# Supplementary material for: Excessive COX‐Prostaglandin Signaling Promotes Cellular Senescence and Impaired Skeletal Muscle Recovery in Aging
Source: Aging Cell. 2026 Nov 13;25(11):e70843. doi: 10.1111/acel.70843 (PMC13768205; doi:10.1111/acel.70843)
Supplement: Supplementary file 4 [file ACEL-25-e70843-s004.pdf]

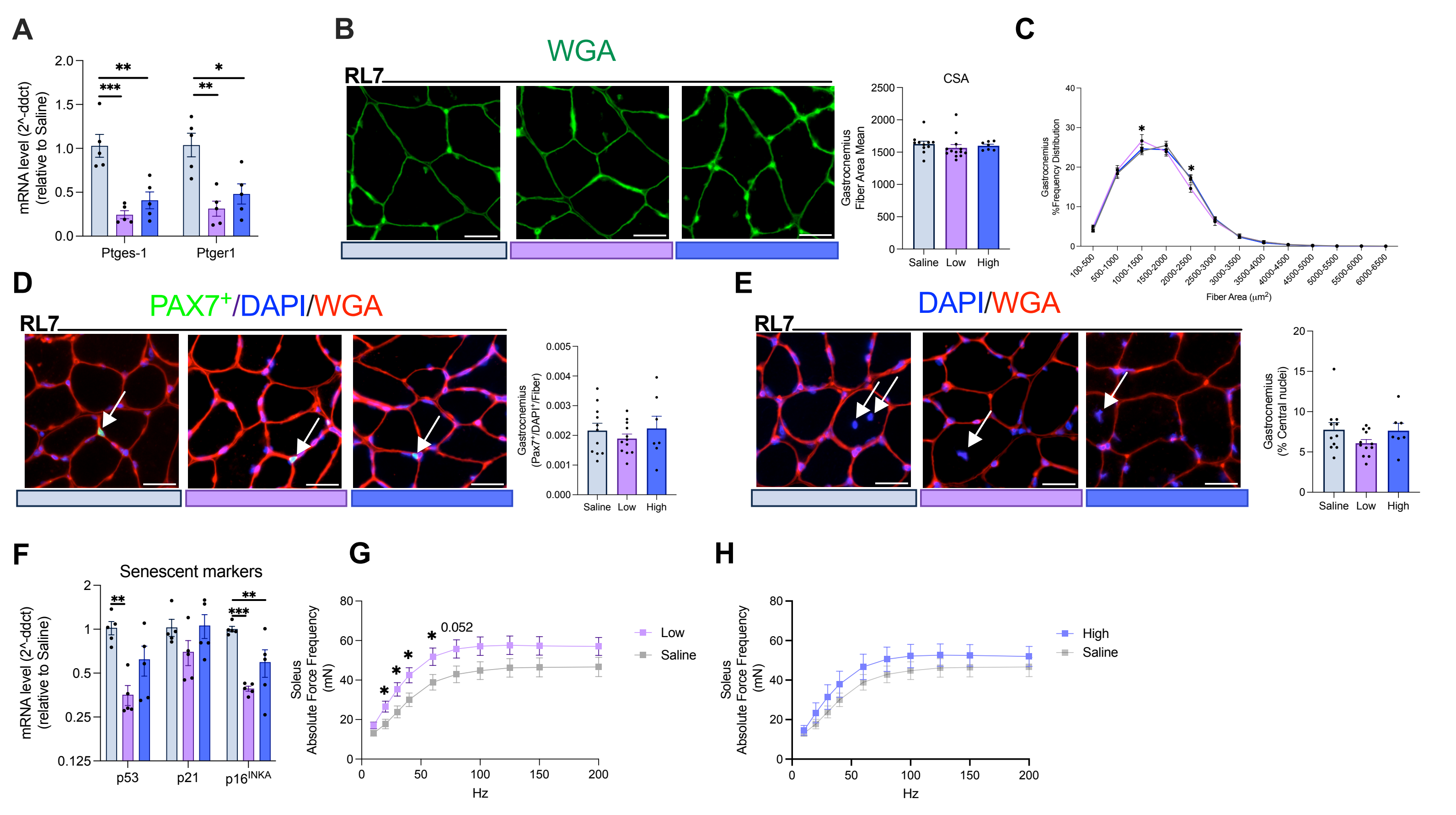

A
mRNA level (2^-ddct) (relative to Saline)
Ptges-1
Ptger1
B
WGA
RL7
CSA
Gastrocnemius Fiber Area Mean
Saline
Low
High
C
Gastrocnemius %Frequency Distribution
Fiber Area (μm2)
D
PAX7+/DAPI/WGA
RL7
Gastrocnemius (Pax7+/DAPI+/Fiber)
Saline
Low
High
E
DAPI/WGA
RL7
Gastrocnemius (% Central nuclei)
Saline
Low
High
F
Senescent markers
mRNA level (2^-ddct) (relative to Saline)
p53
p21
p16INKA
G
Soleus Absolute Force Frequency (mN)
0.052
Low
Saline
Hz
H
Soleus Absolute Force Frequency (mN)
High
Saline
Hz
