# Supplementary material for: Excessive COX‐Prostaglandin Signaling Promotes Cellular Senescence and Impaired Skeletal Muscle Recovery in Aging
Source: Aging Cell. 2026 Nov 13;25(11):e70843. doi: 10.1111/acel.70843 (PMC13768205; doi:10.1111/acel.70843)
Supplement: Supplementary file 5 [file ACEL-25-e70843-s005.pdf]

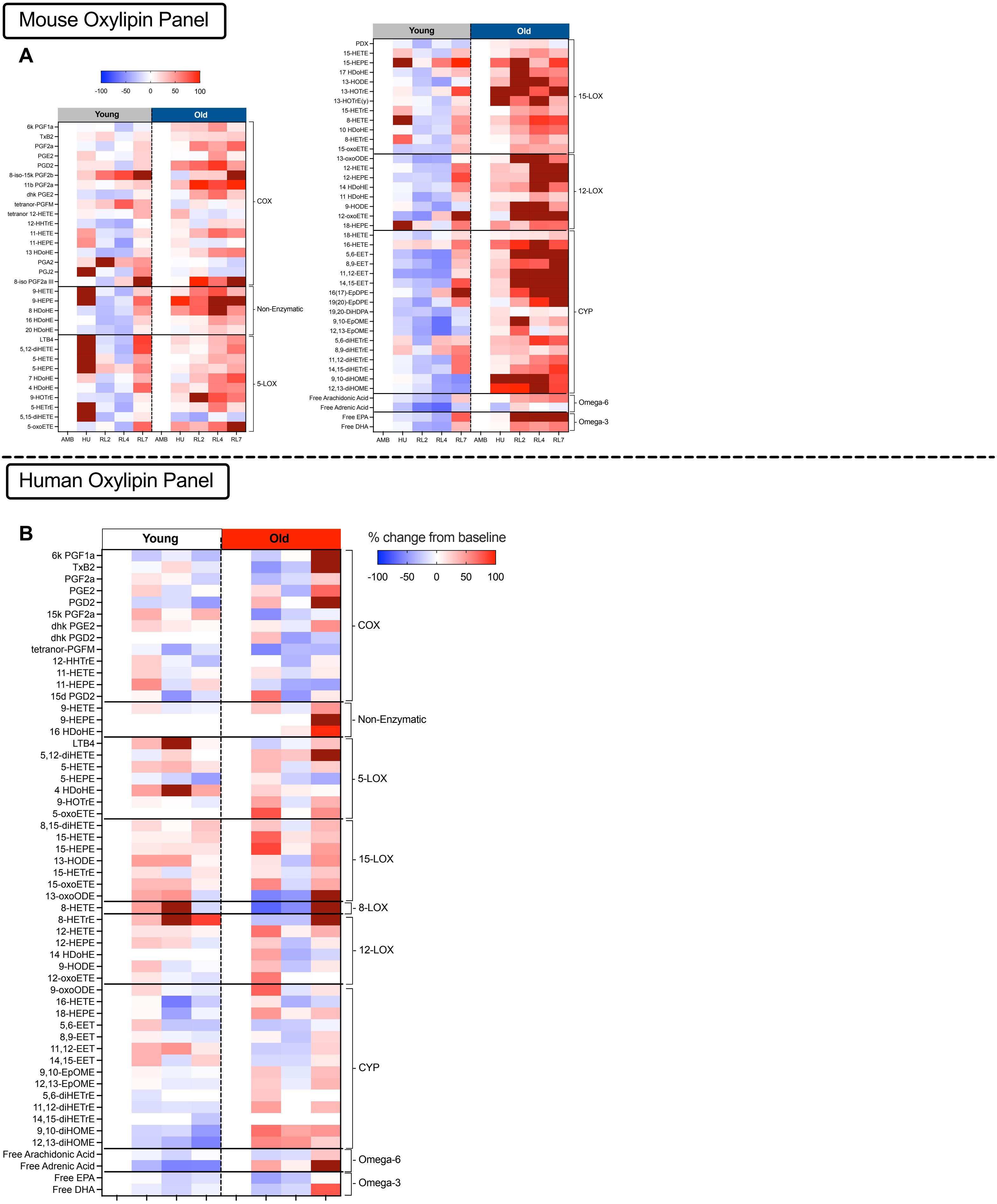

Mouse Oxylipin Panel
A
-100 -50 0 50 100
Young
Old
6k PGF1a
TxB2
PGF2a
PGE2
PGD2
8-iso-15k PGF2b
11b PGF2a
dhk PGE2
tetranor-PGFM
tetranor 12-HETE
12-HHTrE
11-HETE
11-HEPE
13 HDoHE
PGA2
PGJ2
8-iso PGF2a III
COX
9-HETE
9-HEPE
8 HDoHE
16 HDoHE
20 HDoHE
Non-Enzymatic
LTB4
5,12-diHETE
5-HETE
5-HEPE
7 HDoHE
4 HDoHE
9-HOTrE
5-HETrE
5,15-diHETE
5-oxoETE
5-LOX
AMB HU RL2 RL4 RL7 AMB HU RL2 RL4 RL7
Young
Old
PDX
15-HETE
15-HEPE
17 HDoHE
13-HODE
13-HOTrE
13-HOTrE(y)
15-HETrE
8-HETE
10 HDoHE
8-HETrE
15-oxoETE
15-LOX
13-oxoODE
12-HETE
12-HEPE
14 HDoHE
11 HDoHE
9-HODE
12-oxoETE
18-HEPE
12-LOX
18-HETE
16-HETE
5,6-EET
8,9-EET
11,12-EET
14,15-EET
16(17)-EpDPE
19(20)-EpDPE
19,20-DiHDPA
9,10-EpOME
12,13-EpOME
5,6-diHETrE
8,9-diHETrE
11,12-diHETrE
14,15-diHETrE
9,10-diHOME
12,13-diHOME
CYP
Free Arachidonic Acid
Free Adrenic Acid
Omega-6
Free EPA
Free DHA
Omega-3
AMB HU RL2 RL4 RL7 AMB HU RL2 RL4 RL7
Human Oxylipin Panel
B
Young
Old
% change from baseline
-100 -50 0 50 100
6k PGF1a
TxB2
PGF2a
PGE2
PGD2
15k PGF2a
dhk PGE2
dhk PGD2
tetranor-PGFM
12-HHTrE
11-HETE
11-HEPE
15d PGD2
COX
9-HETE
9-HEPE
16 HDoHE
Non-Enzymatic
LTB4
5,12-diHETE
5-HETE
5-HEPE
4 HDoHE
9-HOTrE
5-oxoETE
5-LOX
8,15-diHETE
15-HETE
15-HEPE
13-HODE
15-HETrE
15-oxoETE
13-oxoODE
15-LOX
8-HETE
8-LOX
8-HETrE
12-HETE
12-HEPE
14 HDoHE
9-HODE
12-oxoETE
12-LOX
9-oxoODE
16-HETE
18-HEPE
5,6-EET
8,9-EET
11,12-EET
14,15-EET
9,10-EpOME
12,13-EpOME
5,6-diHETrE
11,12-diHETrE
14,15-diHETrE
9,10-diHOME
12,13-diHOME
CYP
Free Arachidonic Acid
Free Adrenic Acid
Omega-6
Free EPA
Free DHA
Omega-3
Pre Post RL2 RL7 Pre Post RL2 RL7
